# Mapping Phosphorylation-Specific Pin1–CRMP2 Interactions Using an Integrated Mass Spectrometry Approach

**DOI:** 10.1021/acschembio.6c00227

**Published:** 2026-05-26

**Authors:** Danielle F. Kay, Nikolas J. Brooks, Simon G. Caulton, Hiruni S. Jayasekera, Andrew L. Lovering, Aneika C. Leney

**Affiliations:** School of Biosciences, 1724 University of Birmingham, Edgbaston, Birmingham B15 2TT, U.K.

## Abstract

Abnormal protein phosphorylation is a fundamental trigger in the pathogenesis of Alzheimer’s Disease, leading to the formation of neurofibrillary tangles. Thus, molecular determination of the critical factors in controlling phosphorylation is desirable. Pin1, a *cis*–*trans* prolyl isomerase has recently been implicated in Alzheimer’s Disease progression. Moreover, Pin1 specifically targets phosphoproteins, regulating their function. Here, we reveal a novel interaction interface between Pin1 and the Collapsin Response Mediator Protein-2 (CRMP2), a protein found hyperphosphorylated alongside Tau within neurofibrillary tangles. Using native mass spectrometry, we show that Pin1 binds to the disordered C-terminus of CRMP2 in a phosphorylation-dependent manner with residues Thr509 and Thr514 on CRMP2 important for enhanced binding affinity. Hydrogen–deuterium exchange mass spectrometry experiments further localized this binding site to the WW domain of Pin1. Together, these findings provide novel insight into a putative regulatory role of Pin1 in modulating hyperphosphorylation of CRMP2.

## Graphical abstract

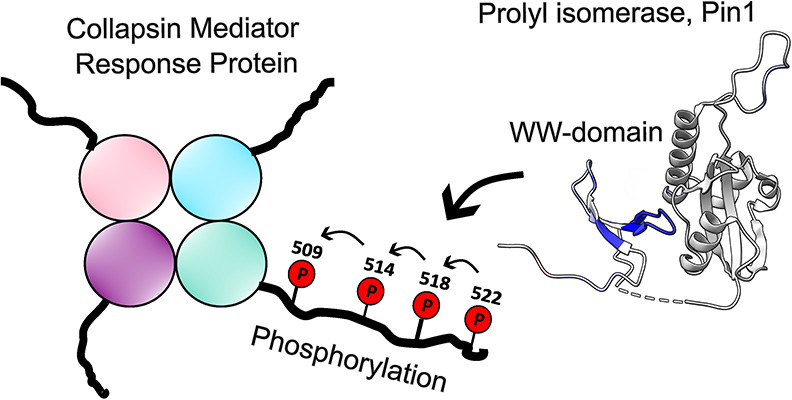

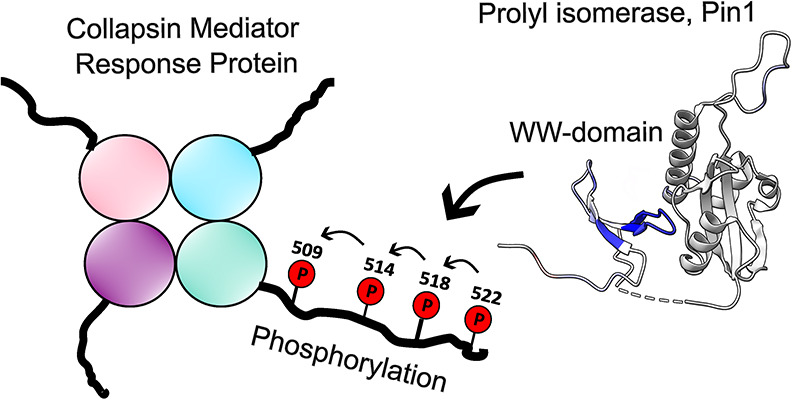

## Introduction

Microtubules are dynamic structures that play important roles in many cellular processes including cell division, cell motility, and intracellular transport. Under normal cellular conditions, microtubule-associated proteins (MAPs) control microtubule function by aiding the transition between their polymerized and depolymerized states.^1^ The function of MAPs is tightly regulated by post-translational modifications (PTMs).^2^ Moreover, modulation of the PTM status of MAPs is known to correlate with cancer^3^ and neurodegenerative diseases including Alzheimer’s and Parkinson’s Disease.^4^

One key MAP whose hyperphosphorylation status has been linked to Alzheimer’s Disease is Tau.^5^ Tau plays an important role in the stability and dynamics of microtubules.^6^ When hyperphosphorylated, Tau can no longer bind to microtubules and promote microtubule assembly. This leads to cytoskeleton destabilization in neurons, and Tau self-aggregation into neurofibrillary tangles that correlates with Alzheimer’s Disease pathology.^7–9^ However, Tau is not the only MAP involved in Alzheimer’s Disease progression. CRMPs are a newly discovered class of MAPs,^10^ wherein CRMP2 specifically is highly expressed in neurons during development and functions to promote microtubule polymerization.^11^ Although CRMP2’s mechanism of action differs from that of Tau, its function is regulated in a similar manner by phosphorylation.^12^ Indeed, elevated levels of soluble, hyperphosphorylated CRMP2 have been detected in the post-mortem brain tissues of both transgenic mice and Alzheimer’s Disease patients^13–16^ making it a possible alternate therapeutic target.

Structurally, CRMP2 is a homotetrameric protein wherein each monomeric unit is composed of a large triosephosphate isomerase (TIM) barrel and small β-sheet domain^17,18^ (Figure 1a). There are two splice variants of CRMP2 (CRMP2A and CRMP2B) that differ in length at their N-terminus.^19^ Both isoforms of CRMP2 also contain identical highly disordered C-terminal tails that are unresolved in the CRMP2B crystal structure yet have proven critical for its role in microtubule attachment.^14^ CRMP2’s C-terminal tails have been found heavily post-translationally modified with both O-glycosylation^20,21^ and phosphorylation^14,22^ detected. Phosphorylation of CRMP2’s C-terminus is sequential, requiring an initial cyclin-dependent kinase-5 (Cdk5)-induced, ‘priming’ phosphorylation event at Ser522, before phosphorylation of the remaining sites by glycogen synthase kinase-3β (GSK-3β)^14,21^ (Figure 1b). C-terminal phosphorylation of CRMP2 reduces the affinity of CRMP2 for tubulin,^12^ thereby promoting microtubule collapse.^23^ Further knowledge on how CRMP2 phosphorylation is controlled may open possibilities for alternative therapeutic intervention. In healthy cells, phosphorylation levels on CRMP2 are dephosphorylated by protein phosphatase-2A.^24^ However, kinases and phosphatases are not the only enzymes that regulate phosphorylation. Many *cis*–*trans* prolyl isomerases (PPIases) act indirectly within cells to control phosphorylation. PPIases can switch proline between its *cis*- and *trans*-isomer within proline-directed kinase and phosphatase substrate motifs that in turn alter the ability of kinases/phosphatases to modify their substrates.^25^ Furthermore, specifically related to CRMP2 regulation, protein phosphatase-2A is conformer-specific, only dephosphorylating pS/T-P motifs when proline is in its *trans*-configuration.^26^ Pin1 (peptidyl-prolyl *cis*–*trans* isomerase, NIMA (never-in-mitosis-A)-interacting 1) is a PPIase, that is unique in that it specifically isomerizes proline at pS/T-P motifs.^27^ Dysregulation of Pin1 activity has been implicated in many MAP-associated diseases including cancer^28^ and Alzheimer’s Disease,^29^ suggesting its likely role in CRMP2-mediated axon guidance. Pin1 possesses a small, 39-residue, WW domain, which is a pS/T-P recognition module, in addition to its PPIase domain.^30^ These two domains are joined by a highly flexible interdomain linker that enables interdomain communication.^31,32^ Pin1 has been shown to bind and facilitate PP2A-mediated dephosphorylation of the MAP Tau at Cdk5 and GSK-3β phosphorylation sites.^26,33^ However, whether Pin1 also interacts with the pS/T-P Pin1 recognition motifs present within the C-terminal tails of CRMP2 is yet to be fully explored.

**Figure 1:**
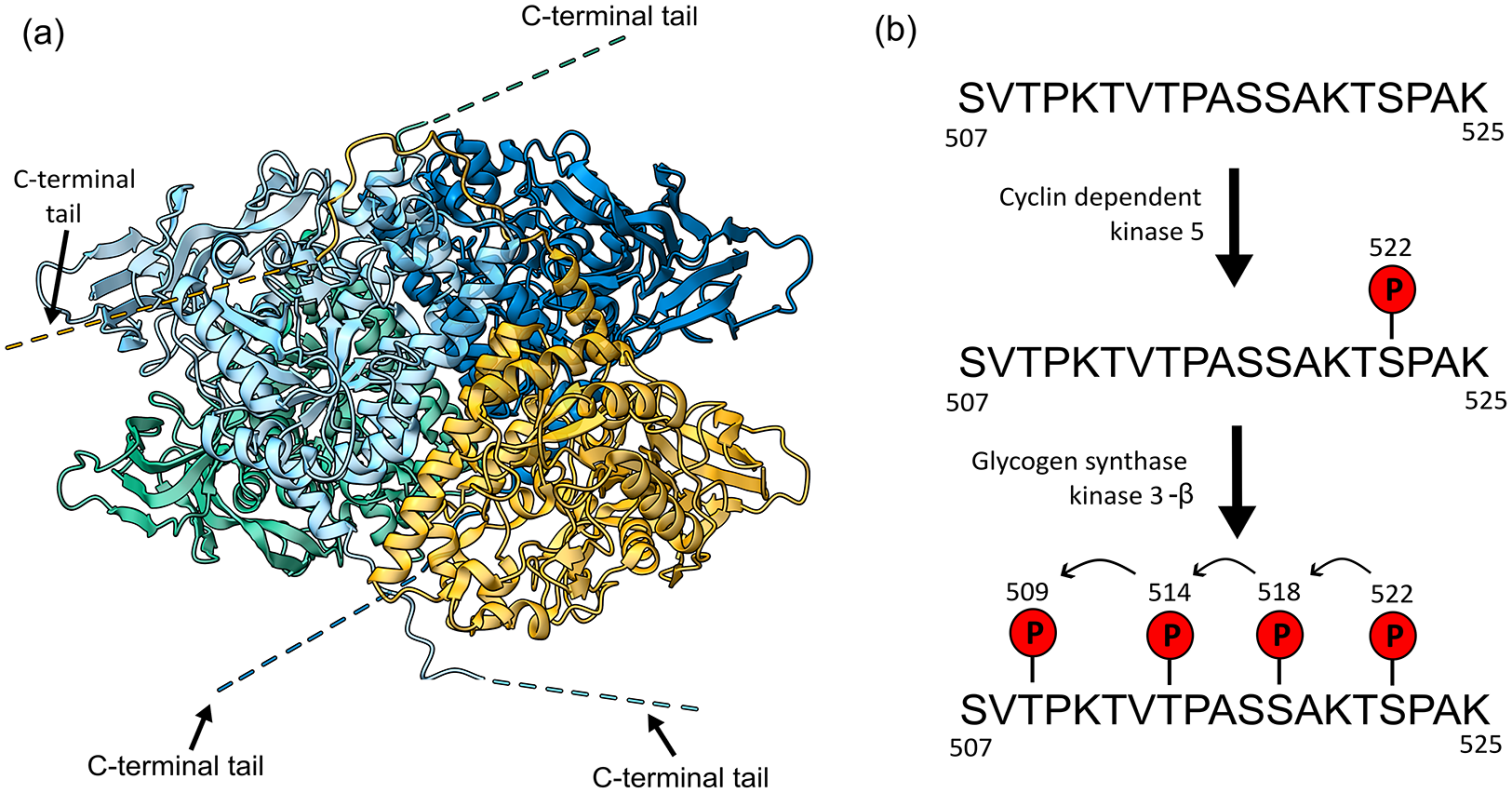
CRMP2 forms a homotetrameric protein complex. (a) CRMP2B_1–496_ tetramer (PDB 6JV9) highlighting the potential location of the disordered C-terminal tails (residues 497–572) of each monomer with an extended dashed line. (b) Size comparison of the Pin1 structure. (c) Schematic showing how each C-terminal tail of CRMP2 becomes sequentially phosphorylated through the activity of Cdk5 and GSK-3β.

Native mass spectrometry (MS) has emerged as an invaluable tool to directly monitor protein interactions. By incubating proteins with ligands of interest and maintaining the interactions they form within the mass spectrometer, the binding stoichiometry and binding equilibria of protein–ligand complexes can be identified.^34–36^ Specific to Pin1, native MS has proven fruitful in its detection of Pin1 interactions with its substrates SMAD3,^37^ the C-terminal domain of RNA polymerase,^37,38^ RNF168,^39^ and cyclic peptide inhibitors.^40^ Furthermore, we have demonstrated that Pin1-mediated interactions can be detected and are dependent on Pin1’s phosphorylation status.^41^ Here, we utilize a native MS approach to investigate whether Pin1 directly interacts with CRMP2’s C-terminal tails and how this interaction occurs. We show that Pin1 binding to CRMP2 is highly dependent upon CRMP2’s phosphorylation status. Moreover, in addition to previously identified Cdk5 phosphorylation sites,^42,43^ we show that phosphorylation by GSK-3β further enhances the affinity of CRMP2 to Pin1 in vitro. By utilizing hydrogen–deuterium exchange mass spectrometry (HDX-MS), we map the binding interface of CRMP2 to Pin1’s N-terminal WW domain. Overall, the data suggests a recruitment mechanism wherein Pin1 binds in concession to multiple phosphorylation sites across CRMP2’s C-terminus, acting as a putative regulatory modulator of hyperphosphorylation within healthy neurons.

## Results

### Pin1 Binds Hyperphosphorylated CRMP2

Previous studies using a pull-down proteomics approach have shown that Pin1 binds predominantly to phosphorylated S27 on the N-terminus of CRMP2A with data suggesting that S522 may also be involved.^43^ This finding was based on the differential binding of CRMP2A and CRMP2B (that differ in their N-termini) to Pin1 within SH-SY5Y cells during mitotic arrest induced by Nocodazole.^43^ Under these conditions, Cdk5-mediated phosphorylation sites are the most abundant phosphorylation sites on CRMP2, with GSK-3β-mediated phosphorylation sites lacking due to Nocodazole’s indirect ability to inhibit GSK-3β activity. Therefore, we first set out to determine whether the unstructured hyperphosphorylated C-terminus of CRMP2 could be an additional site of direct interaction with Pin1 that further enhances its interaction affinity. Peptides corresponding to residues 507–525 of CRMP2 (herein termed CRMP2_507–525_) were synthesized whereby S/T residues were differentially phosphorylated corresponding to CRMP2’s proteoforms observed in vivo^14^ (Figure 1b, Table 1), and their interactions with Pin1 probed by native MS. Native MS analysis of Pin1 alone shows a narrow charge state distribution corresponding to a mass of 18,313 Da (Figure 2a, Table S1). Upon addition of unmodified CRMP2_507–525_, no Pin1–CRMP2_507–525_ complex was observed (Figures 2b and S1). This is consistent with Pin1’s unique role among PPIases in binding specifically to phosphorylated proteins. Next, Pin1 was incubated with CRMP2_507–525_ pS522, whereby pS522 was used to mimic Cdk5-induced phosphorylation of CRMP2 (Figure 2c). Low abundance peaks were observed corresponding to a 1:1 complex of Pin1:CRMP2_507–525_ pS522. Interestingly, upon addition of the sequentially phosphorylated CRMP2_507–525_ peptides, used to mimic stepwise GSK-3β-induced phosphorylation on CRMP2, complex formation increased (Figure 2d–f) with up to 30% of Pin1 observed bound to the quadruply phosphorylated CRMP2 peptide in a 1:1 complex (Figure 2f). The extent of binding observed is higher than the known interaction of Pin1 with pS27 on the N-terminus of CRMP2A (Figure S2a, Table S1). Moreover, the binding affinity observed is consistent with other known Pin1 interaction sites such as pT48 on Cdc25C (Figure S2b) and pS235 or pT231pS235 on Tau (Figure S2c,d).

**Table 1:** Complex Formation of Pin1 with Differentially Phosphorylated C-Terminal Tails of CRMP2^a^

peptide name	CRMP2 peptide sequence	% complex formation
CRMP2_507–525_	_507_SVTPKTVTPASSAKTSPAK_525_	1 ± 0.4
CRMP2_507–525_ pS522	_507_SVTPKTVTPASSAKT**S**PAK_525_	2 ± 0.3
CRMP2_507–525_ pS518, pS522	_507_SVTPKTVTPAS**S**AKT**S**PAK_525_	5 ± 2
CRMP2_507–525_ pT514, pS518, pS522	_507_SVTPKTV**T**PAS**S**AKT**S**PAK_525_	25 ± 7
CRMP2_507–525_ pT509, pT514, pS518, pS522	_507_SV**T**PKTV**T**PAS**S**AKT**S**PAK_525_	32 ± 2
CRMP2_512–520_ pT514	_512_TV**T**PASSAK_520_	11 ± 0.1
CRMP2_512–520_ pT514, pS518	_512_TV**T**PAS**S**AK_520_	6 ± 4
CRMP2_507–517_	_507_SVTPKTVTPAS_517_	<1
CRMP2_507–517_ pT514	_507_SVTPKTV**T**PAS_517_	9 ± 0.4
CRMP2_507–517_ pT509	_507_SV**T**PKTVTPAS_517_	9 ± 2
CRMP2_507–517_ pT509, pT514	_507_SV**T**PKTV**T**PAS_517_	26 ± 4

a Phosphothreonine and phosphoserine residues are highlighted in black. % complex formation was measured at a 1:4 ratio Pin1:CRMP2 peptide.

**Figure 2:**
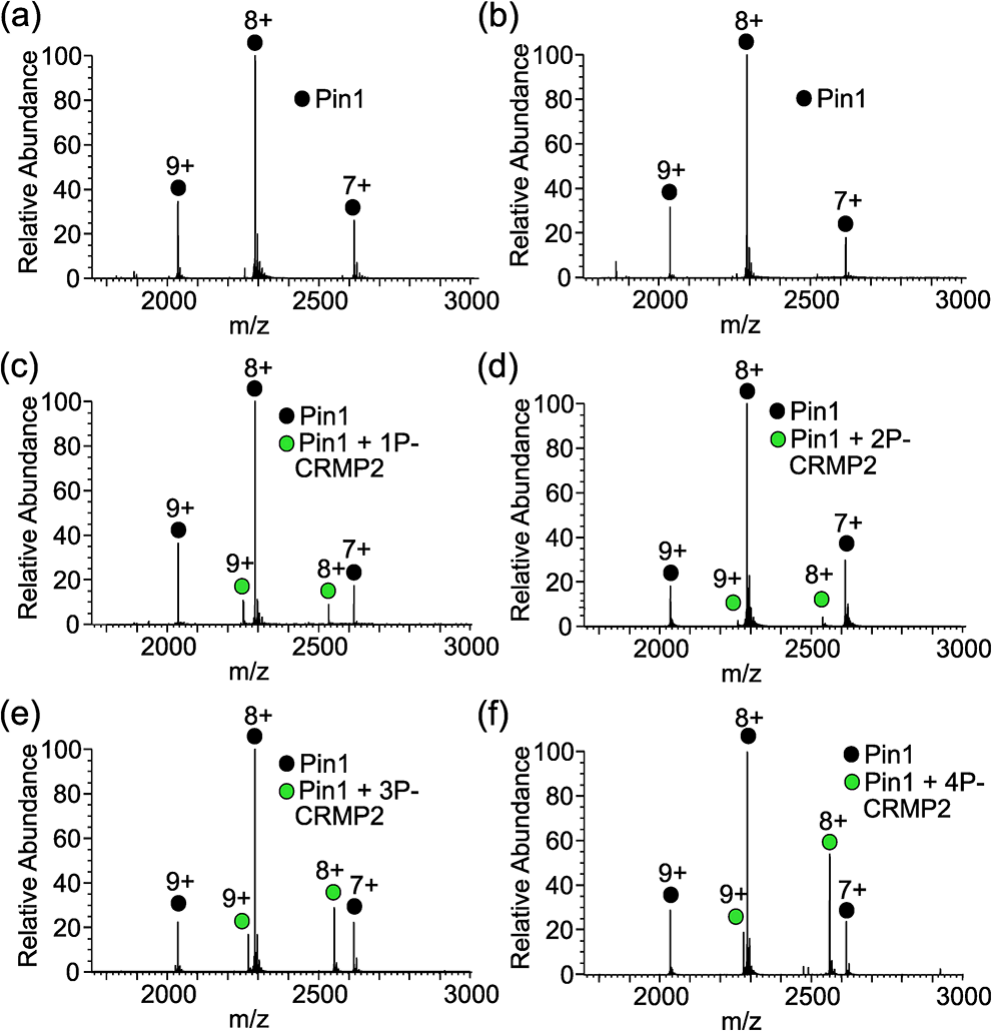
Native MS shows Pin1 preferentially binds the hyperphosphorylated C-terminus of CRMP2_507–525_. Pin1 (5 μM) in the absence (a) and presence of 20 μM CRMP2 (b), CRMP2 pS522 (c), CRMP2 pS518, pS522 (d), CRMP2 pT514, pS518, pS522 (e), and CRMP2 pT509, pT514, pS518, pS522 (f). Peaks highlighted in green indicate Pin1–peptide complex species formed. Peaks corresponding to unbound Pin1 protein are shown in black.

### Thr509 and Thr514 Are Dominant Recruitment Sites on CRMP2 for Pin1

Due to the length of the hyperphosphorylated CRMP2’s C-terminus compared with the Pin1 domains (Figure S8) and the proximity of the pT509, pT514, pS518, and pS522 residues, we hypothesized that all phosphorylation sites may not be contributing equally to its binding affinity for Pin1. Thus, we next synthesized shorter peptides, CRMP2_512–520_ and CRMP2_507–517_, with differential GSK-3β phosphorylation sites (Table 1) to determine which phosphorylation sites are critical to the Pin1 interaction. Upon incubation of CRMP2_512–520_ containing the two phosphorylation sites pT514 and pS518 with Pin1, minimal complex formation was observed (Figure 3a, Table 1). In contrast, a large peak corresponding to a 1:1 complex was observed between Pin1 and CRMP2_512–520_ when T509 and T514 were phosphorylated (Figure 3b). Considering the sequential order of GSK-3β-induced phosphorylation along the CRMP2 tails from its C-terminus to its N-terminus, this suggests that Pin1 binds the CRMP2 C-terminal tails only when all possible phosphorylation sites are phosphorylated, i.e., when CRMP2 is hyperphosphorylated. Moreover, both pT514 and pT509 contribute to this high affinity interaction suggesting that these are the dominant sites for Pin1 recruitment. Reduced binding was observed for individual phosphorylated CRMP2 peptides (Figures 3c,d and S3). Importantly, no peaks are observed corresponding to a 2:1 CRMP2:Pin1 complex, hinting that these phosphorylation sites on CRMP2 either bind independently to different pS/T-recognizing domains, or more likely, they are competing for the same Pin1 binding site. Indeed, due to the small size of Pin1 (18 kDa) related to the intact CRMP2 tetramer (64 kDa), up to four molecules of Pin1 could be binding to CRMP2 in vivo, one to each C-terminal tail within the CRMP2 tetramer.

**Figure 3:**
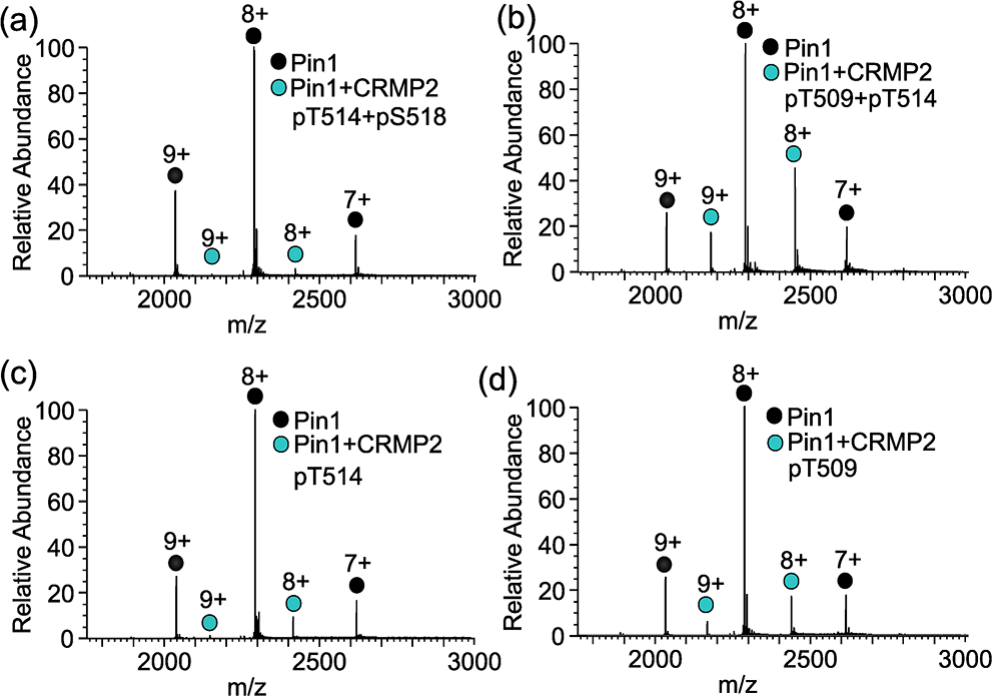
Native MS shows dual phosphorylation at Thr509 and Thr514 on CRMP2 which are dominant sites for Pin1 binding. Pin1 (5 μM) was incubated with 20 μM CRMP2_512–520_ pT514, pS518 (a), CRMP2_507–517_ pT509, pT514 (b), CRMP2_512–520_ pT514 (c), and CRMP2_507–517_ pT509 (d). Peaks highlighted in blue indicate Pin1–peptide complex species, while unbound Pin1 protein peaks are colored black.

To confirm Pin1 interacts with hyperphosphorylated CRMP2 in solution, isothermal titration calorimetry (ITC) experiments were carried out with CRMP2_507–517_ with and without pT509 and pT514 (Figure 4a). Consistent with native MS analysis, binding to CRMP2_507–517_ was found to be phospho-dependent (Figure 4a). An overall 1:1 binding stoichiometry (*N* = 0.77 ± 0.05) was observed with an approximate affinity of 82 μM ± 15 μM between Pin1 and CRMP2_507–517_ pT509 and pT514 while no binding was observed with unmodified CRMP2_507–517_. This affinity is consistent with other known Pin1 substrates such as Cdc25 (117 μM)^44^ and Tau (90–180 μM).^33,45^

**Figure 4:**
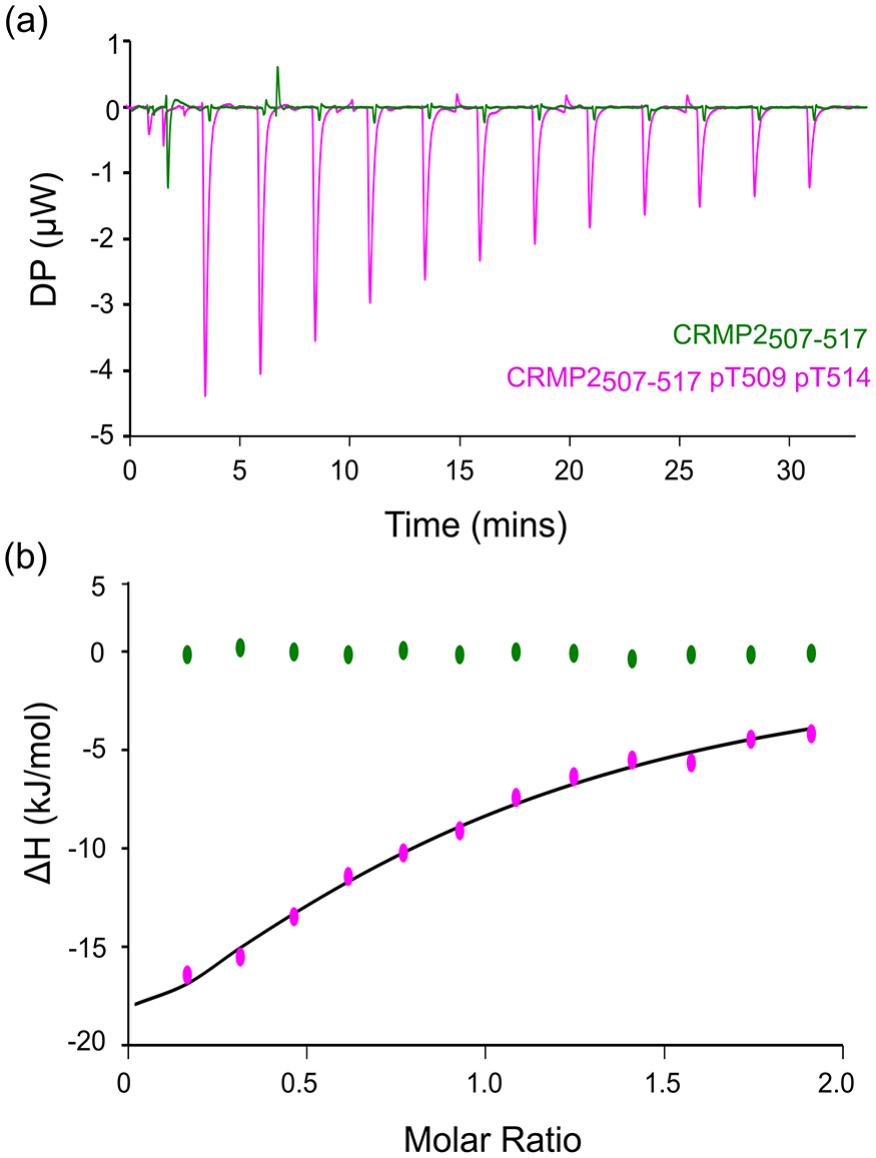
ITC confirms CRMP2’s C-terminus binds Pin1 in a phosphorylation-dependent manner. 1 mM CRMP2_507–517_ (green) or CRMP2_507–517_ pT509, pT514 (pink) were titrated into Pin1 (100 μM). Overlaid thermograms (a) and binding isotherms (b) are shown, where binding is only observed for the CRMP2_507–517_ pT509, pT514.

### CRMP2–Pin1 Interaction Localized to the WW Domain of Pin1

To localize the CRMP2 binding site on Pin1, bottom-up HDX-MS was carried out. HDX-MS is a valuable tool in structural biology that involves measuring protein dynamics. Complementary to native MS, HDX-MS can reveal insight into ligand binding sites and binding-induced structural changes.^46–48^ To perform HDX-MS, Pin1 was incubated alone and in the presence of either CRMP2_507–517_ or CRMP2_507–517_ pT509, pT514 in deuterated buffer for 30 s, 10 min, and 100 min. The reaction was quenched, the protein digested, and the deuterium uptake at the peptide level analyzed by liquid chromatography (LC) coupled with MS. This yielded 113 peptide identifications shared across all incubation conditions and deuteration time points, corresponding to 96% sequence coverage (Figure S4). In apo Pin1, the extent of deuterium labeling differed across the Pin1 sequence (Figure S5). The WW domain exhibited high deuterium labeling, indicating a region of high conformational dynamics. The PPIase domain exhibited a greater range in deuterium labeling with some regions having <40% labeling at 100 min indicative of a more stable and compact structure. Upon addition of unphosphorylated CRMP2_507–517_, no change in deuterium labeling was observed across the Pin1 sequence (Figures S6 and S7), consistent with the lack of binding observed by native MS (Figures 2b and S1) and ITC (Figure 4).

Pin1 is known to bind proline-containing phosphopeptides in two regions. On the PPIase domain, L122, M130, and F134 interact with the proline residue of substrates while K63, R68, and R69 accommodate the phosphorylated residue of substrates.^27^ On the WW domain, residues S16–R21 on the first loop contribute to a high affinity interaction,^49,50^ while Y23–W34 aids the binding of the *trans*-isomer of proline (Figure S8).^50^ Upon Pin1–CRMP2_507–517_ pT509 pT514 complex formation, a reduction in deuterium uptake was observed on peptides spanning the N-terminal WW domain of Pin1, with the highest level of protection visible after 30 s (Figures 5a,b, S6, and S9). The loss of protection at later time-points compared to apo Pin1 is likely due to the transient nature of the Pin1–CRMP2 interaction. Moreover, during the HDX reaction, apo Pin1 and the Pin1–CRMP2 complex were present in equilibrium. Protected regions from deuterium labeling at 30 s were mapped onto the X-ray structure of Pin1 bound to the doubly phosphorylated C-terminal domain of RNA polymerase II,^50^ which highlighted the confinement of protection to the WW domain (Figure 5c). As predicted based on all phosphopeptide-bound Pin1 structures, the residues S16, Y23, F25, and S32 on Pin1’s WW domain are within the regions protected from deuterium exchange (Figures 5c, S8, and S9). No protection was observed on peptides covering the PPIase binding catalytic loop (Figures 5b and S10). Subtle variations in deuterium uptake were observed on residues 78–85 and 92–97 (Figure S9), hinting that either CRMP2 phosphopeptide is binding with lower affinity to the PPIase domain and is a substrate of Pin1 or that allosteric interactions are occurring between the WW domain and PPIase domain of Pin1 upon CRMP2 binding. In support of this, previous studies on Pin1 have shown a complex interdomain communication system exists whereby prolyl *cis*–*trans* isomerization on Pin1 substrates can either be increased or decreased dependent on their WW-domain binding mode.^51–54^

**Figure 5:**
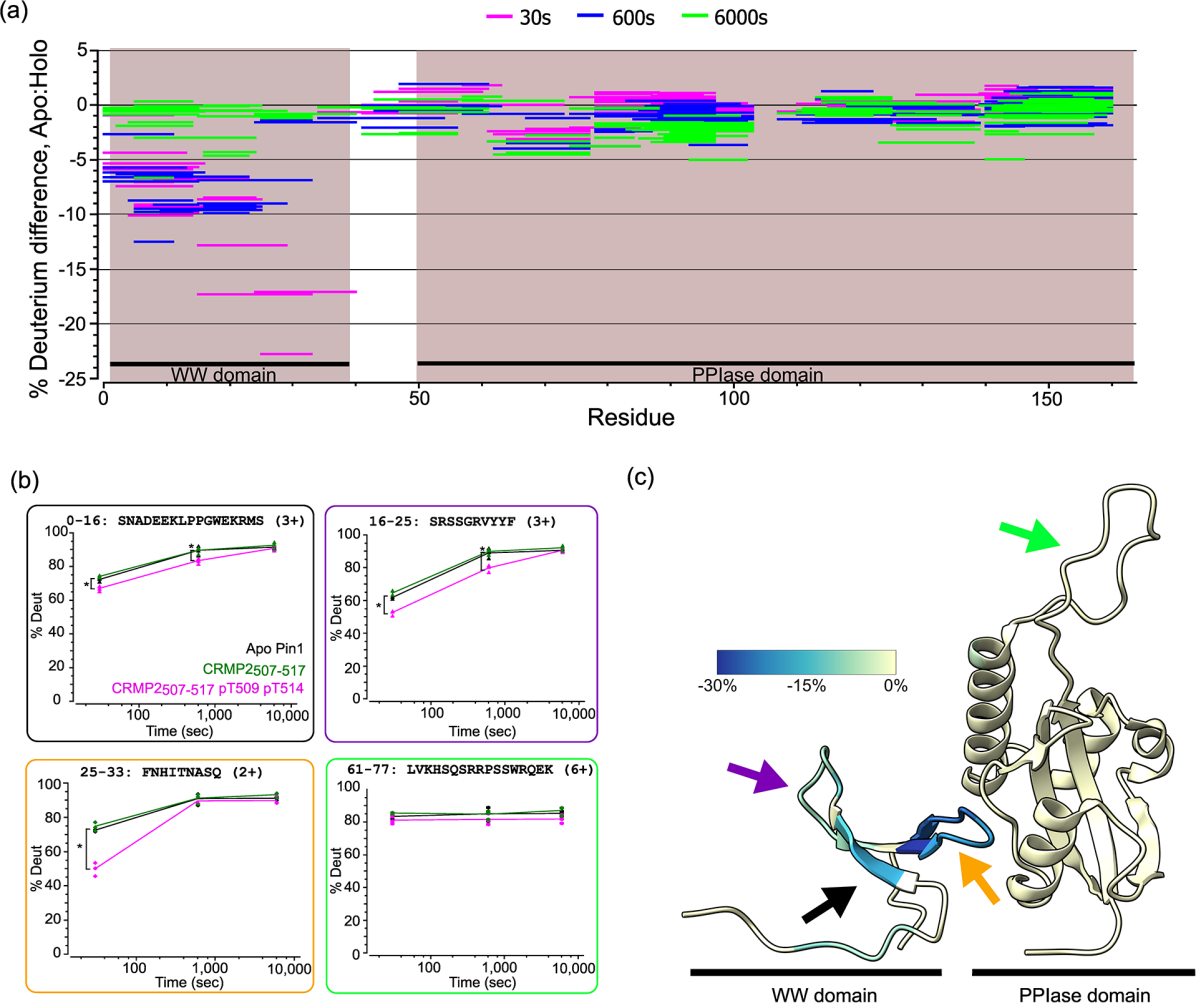
Pin1/CRMP2_507–517_, pT509, pT514 binding interaction revealed by HDX-MS. (a) Woods plot highlighting the difference in deuterium uptake between apo Pin1 and Pin1 incubated with CRMP2_507–517_ pT509, pT514 on peptides across the Pin1 sequence. Horizontal lines represent each peptide detected by LC-MS. The lines are colored pink, blue, and green according to the time incubated in deuterium, 30 s, 600 s, and 6000 s, respectively. (b) Representative uptake plots of four peptides of Pin1 displaying % deuteration incorporation in the apo (black), Pin1/CRMP2_507–517_ pT509, pT514 bound (pink), and Pin1/CRMP2_507–517_ bound (green) condition. (c) HDX difference profile at 30 s mapped onto the Pin1 structure (PDB: 1f8a). The arrows correspond to where the peptides from (b) are present on the Pin1 structure.

## Discussion

CRMP2 is a MAP whose hyperphosphorylation status in cells is critically controlled. Previous work has shown that in developing neurons, phosphorylation of CRMP2 by Cdk5 at S27 and S522 creates canonical binding motifs for Pin1 leading to stabilization of CRMP2.^43^ Further regulation of CRMP2’s unphosphorylated form by the Pin1-related PPIase, FK509, has also been observed, acting as a negative regulator of microtubule dynamics and axon growth.^55^ Here, using native MS, we show that Pin1 additionally binds directly to the C-terminal phosphorylated sites of CRMP2 in vitro. Furthermore, we localize this dominant binding interface to be between pT509 and pT514 sites on CRMP2 and the WW domain of Pin1.

Pin1 interacts with a plethora of biological substrates,^56–58^ some of which contain a single pS/T-P motif, and others contain multiple motifs spaced differentially apart.^59^ As such, several distinct binding models have been proposed.^60^ The most widely accepted Pin1 binding model is the sequential binding model, whereby the protein containing the pS/T-P motif first interacts with Pin1’s WW domain, bringing the Pin1 PPIase domain in proximity to interact with the same or an alternative pS/T-P site present on the same target substrate. Our native MS studies show that when Pin1–CRMP2 complexes are observed, they form with a 1:1 stoichiometry. This is consistent with Pin1’s 10-fold higher affinity for the WW domain compared with the PPIase domain.^50^ Indeed, due to their transient nature, enzyme–substrate reactions are rarely observed by native MS. Importantly, we show that even when two phosphosites are present on the same CRMP2 peptide, although the affinity of the interaction with Pin1 increases, a 1:1 complex remains. Our data suggests that pT509 and pT514 on CRMP2 are the dominant recruitment sites for Pin1 within the C-terminal tails (Figure 3); however, other C-terminal phosphosites such as pS522 also bind to some extent (Figure 2c). The increase in the bound complex when comparing the single CRMP2_507–517_ pT509 and CRMP2_507–517_ pT514 sites with the doubly phosphorylated CRMP2507–517 pT509, pT514 (Figure 3) is intriguing. A similar observation has also been observed with other Pin1 substrates, one example being the C-terminal domain of RNA polymerase II, whereby the doubly phosphorylated peptide showed enhanced affinity over its singly modified counterparts.^50^ The binding of multiple pSP/pTP sites in CRMP2’s C-terminus suggests a ‘sliding’ mechanism may exist whereby the Pin1 substrate is not fully released and instead slides across the Pin1 protein to enhance its local concentration and prevent competitive interactors (e.g., phosphatases) from accessing these important phosphosites until the isomerization reaction has occurred. No further increase in complex abundance was observed upon addition of the pS518 phosphosite (Figure 3a, Table 1) showing that phosphorylation by GSK-3β is critical for the high affinity interaction of CRMP2’s C-terminus with Pin1, with proline being adjacent to S/T important in the Pin1 recognition site.

Since binding of the pT509 and pT514 CRMP2 residues has been localized to the WW domain and is too close in proximity to bind both binding sites of Pin1’s WW domain and PPIase domain simultaneously, we hypothesize that these sites are dominant sites on CRMP2 for Pin1 recruitment. AlphaFold3 studies on the intact Pin1–CRMP2 complex showed Pin1 interacts directly with the phosphorylation sites on the CRMP2’s C-terminal tails as opposed to pSer27; however, it should be noted that the template modeling scores are very low due to the unstructured nature of CRMP2’s termini, resulting in low confidence on the interactions sites within these structural predictions (Figure S12). Whether the adjacent prolines to CRMP2’s C-terminal phosphosites are substrates for Pin1’s PPIase domain remains to be deciphered. Assuming the hyperphosphorylated CRMP2 C-terminus remains bound while proline isomerization occurs, the isomerization site is unlikely to be pS522-P since in all known Pin1–peptide binding structures the C-terminal end of the bound peptide extends away from the center of Pin1, in the opposite direction from the PPIase domain (Figure S8). Consistent with this observation, Sutherland and co-workers showed Pin1 had no effect on phosphorylation or dephosphorylation of the CRMP2B isoform.^42^ A more likely isomerization site is the previously identified pS27-P site on the flexible N-terminus of CRMP2 which we have also shown binds Pin1 (Figure S2a) but to a lesser extent. Consistent with this, negligible/very subtle changes in deuterium labeling were observed within the interdomain region of Pin1, suggesting that once CRMP2 is bound, Pin1 is still in a flexible state to be able to move and reach alternative proline sites, rather than having its conformational dynamics reduced. In a similar manner, Pin1 has previously been found to bind multiple pS/T-P motifs on Tau but not catalyze the isomerization of all of these motifs.^51^

## Conclusion

Overall, we show Pin1 recognizes GSK3β-induced phosphorylation motifs in addition to its previously identified cdk5-induced phosphorylation sites on CRMP2 through its high-affinity WW domain. Although precisely which proline residues within CRMP2 undergo *cis*–*trans* isomerization remains to be deciphered, we hypothesize that in vivo once hyperphosphorylated CRMP2 binds, it facilitates the PPIase domain of Pin1 to act rapidly on other pS/T-P isomerization sites within CRMP2 regulating its function. Thus, while further in-cell and in vivo research is needed into why Pin1 interacts with GSK3β-induced CRMP2 phosphorylation motifs, our work provides fruitful insight into the complexity behind how post-translational modifications on CRMP2 act in protein recruitment to regulate their overall function.

## Materials and Methods

### Pin1 Expression and Purification

The Pin1-his_6_ plasmid was gifted from Dustin Maly (Addgene no. 40773) and transformed into *E. coli* BL21 (DE3) cells (New England BioLabs). Pin1His6 was expressed recombinantly and purified using Ni-affinity chromatography. His-tagged TEV protease was added at a 50:1 Pin1:TEV protease ratio to cleave the His6 tag. The uncleaved Pin1His6 and TEV protease were removed using Ni-affinity chromatography, and the final Pin1 construct was further purified using size exclusion chromatography. The purity of the final Pin1 construct was confirmed using SDS-PAGE and mass spectrometry analysis, and its activity verified using a chymotrypsin-coupled Pin1 PPIase assay.^61^ Pin1 was stored in 50 mM HEPES, 300 mM NaCl, and 1 mM DTT, pH 7.4 at −80 °C until further use.

### Synthetic Peptides

Peptides corresponding to residues 507–525, 507–517, and 512–520 of intact human CRMP2, with varying phosphorylation patterns, were synthesized and purchased from Synpeptide Co., Ltd. (Shanghai, China), with >95% purity. Peptides incorporating residues 512–520 and 507–517 were N-terminally acetylated and C-terminally amidated. The sequences, phosphorylation sites, and corresponding molecular masses are presented in Table S1. 100% phosphosite occupancy was confirmed on the phosphopeptides corresponding phosphorylation sites by mass spectrometry. The lyophilized peptides were dissolved into 50 mM ammonium acetate pH 6.8 and stored at a concentration of 100 μM at −20 °C prior to use.

### Native Mass Spectrometry

For native MS analysis, Pin1 was buffer exchanged into a 50 mM ammonium acetate pH 6.8 solution by consecutive dilution and concentration steps using 3 kDa molecular weight cutoff (MWCO) Amicon Ultra 0.5 mL centrifugal filters. Pin1 was mixed with CRMP2 peptides (Tables 1 and S1) at a 1:4 Pin1:CRMP2 ratio (5 μM:20 μM) in 50 mM ammonium acetate pH 6.8. All reaction mixtures were allowed to equilibrate at RT for approximately 5 min before native MS analysis. For timed-incubation experiments that mimic HDX-MS conditions, Pin1 was incubated with CRMP2_507–517_ pT509, pT514 at a 1:10 Pin1:CRMP2 ratio (5 μM:50 μM) in 50 mM ammonium acetate pH 6.8 for ∼1, 10, and 100 min, and the mass spectra acquired immediately.

All native MS experiments were conducted on a Q-Exactive HF mass spectrometer (Thermo Fisher Scientific) coupled to either a TriVersa NanoMate nanoflow-electrospray ionization source (Advion Biosystems Inc.) or a nanoelectrospray ionization source that used gold-coated borosilicate glass capillaries, pulled in-house. Positive ion mode was used and the capillary voltage was set between 0.75 and 1.2 kV. The in-source fragmentation was set to zero, source temperature 250 °C, and S-lens RF 100. A mass range of 400–4000 *m*/*z* was used throughout. Mass spectra were scanned with a maximum ion injection time of 100 ms and automatic gain control of 1 × 10^6^. Ions were detected in the Orbitrap with the resolution set to 15,000 at *m*/*z* 200.

All mass spectra were processed by using XCalibur v4.3 (Thermo Fisher Scientific). For % complex formation, the relative peak intensities of the Pin1–peptide complex were summed and divided by the sum of the peak intensities for both Pin1 alone and any Pin1–peptide complex observed ×100%. A minimum of two replicates were performed for each complex. The average % complex formation was reported alongside the standard deviation among all replicates.

### Isothermal Titration Calorimetry

ITC was performed using a MicroCal PEAQ-ITC. Pin1 was buffer exchanged against 20 mM HEPES, 100 mM NaCl, pH 7.4 using 3 kDa MWCO Amicon Ultra centrifugal filters. Lyophilized CRMP2 peptides (CRMP2_507–517_ and CRMP2_507–517_ pT509, pT514) were dissolved in 20 mM HEPES, 100 mM NaCl, pH 7.4 to a final concentration of 1 mM. 1 mM of each CRMP2 peptide was loaded into the syringe and titrated into 100 μM Pin1 in the cell. The titration consisted of 13 injections with an initial injection of 0.4 μL over 0.8 s, followed by 12 injections of 3 μL over 6 s, with 150 s spacing between every injection. The cell was set to 25 °C, with a stirring speed of 750 rpm. Control experiments of 1 mM CRMP2_507–517_ or CRMP2_507–517_ pT509, pT514 into 20 mM HEPES, 100 mM NaCl, pH 7.4 showed negligible heats and were subtracted from their corresponding assay prior to single-site modeling of the curve using MicroCal PEAQ-ITC software v1.41.

### Hydrogen–Deuterium Exchange Mass Spectrometry

Pin1 (50 μM) was incubated alone and in both the presence of the unphosphorylated CRMP2_507–517_ and doubly phosphorylated CRMP2_507–517_ pT509, pT514 peptides (500 μM). For HDX, either Pin1 or the Pin1 complexes were diluted 10-fold in 50 mM ammonium acetate 99.9% D_2_O pD 7.2 for 30 s, 10 min, and 100 min. Maximally deuterated samples were prepared in triplicate by diluting Pin1 1:1 with 4 M urea, 200 mM potassium phosphate buffer pH 2.4. The sample was then lyophilized before resuspending into 50 mM ammonium acetate 99.9% D_2_O pD 7.2 and incubated for 72 h. All Pin1 samples were quenched in 4 M urea, 200 mM potassium phosphate buffer, pH 2.4, followed by flash freezing in liquid nitrogen and stored at −80 °C. Samples were thawed and 2.2 μg of protein immediately injected onto a home-built, online HDX-MS setup (Figure S13). The samples were injected through valve 1 onto a dual nepenthesin-2/pepsin POROS column (2.1 mm × 20 mm, 7 °C, Affipro) at a flow rate of 0.1 mL/min (MX-Class Auxiliary Pump, Thermo Fisher Scientific) (ice-cold 0.1% formic acid in the water mobile phase) and the digested peptides collected on a C18 trap column (ACQUITY UPLCBEH 1.7 μm, 2.1 mm × 5 mm, Waters). After 3.5 min, the valves were switched and the peptides eluted and separated with a C18 analytical column (Hypersil GOLD Vanquish, 50 mm × 2.1 mm, 1.9 μm, Thermo Fisher Scientific) connected to a vanquish UHPLC pump (Thermo Fisher Scientific). A flow rate of 350 μL/min was used with mobile phase A of H_2_O and 0.1% formic acid and mobile phase B of 100% acetonitrile and 0.1% formic acid. A gradient from 4% to 12% B over 0.3 min and from 12% to 40% B over 5.2 min was used for peptide elution. Peptide masses were analyzed on an Orbitrap Eclipse Tribrid mass spectrometer coupled to an electrospray ionization source (Thermo Fisher Scientific). Positive ionization mode was used throughout, with the capillary voltage set to 3.5 kV. The HESI source temperature was set at 250 °C, vaporizer temperature at 50 °C, and S-lens RF at 60. For Pin1 peptide identification, precursor ions were selected based on the top 10 abundant ions, isolated in the quadrupole using a 2 *m*/*z* window, and fragmented using a normalized higher-energy collisional dissociation (HCD) energy of 30. Fragment ions were detected in the Orbitrap using a resolution setting of 30,000. Dynamic exclusion was employed for 4 s on a single-charge state per precursor, and only charge states from 2+ to 10+ were selected for MS/MS. For deuterated samples, full scan MS1 spectra were acquired in the Orbitrap mass analyzer using a resolution of 60,000 and a mass range between 255 and 2000 *m*/*z*. The maximum injection time and automatic gain control were set to auto. All time points were analyzed in triplicate. Two blank (water and 0.1% formic acid) injections in-between protein injections ensured the absence of sample carryover.

### Hydrogen–Deuterium Exchange Mass Spectrometry Data Analysis

Pin1 peptides were validated using Protein Discoverer v2.5. (Thermo Fisher Scientific). For all searches, enzyme cleavage was set to semi, and the maximum number of missed cleavages set to 12 with a minimum peptide length of 4. A precursor mass tolerance of 10 ppm was used, with a fragment mass tolerance of 0.02 Da. The peptide validator node was set to filter for a false discovery rate of 0.01. Only peptides detected in all 3 replicates and in all conditions were reported. The peptide list and corresponding retention times were exported and subsequent data analysis carried out in HDExaminer (v3.4.2, Sierra Analytics, Modesto, CA) (Tables S3 and S4).

Deuterated peptides were filtered based on their matches to the theoretical isotopic distribution, consistent with overlapping peptides and charge states and spectrum crowding. Deuterated peptides with >20 residues were excluded from analysis. The back-exchange within the HDX device was calculated to be 32% (ranging from 21 to 48% between experimental setup) using a sample of 18 peptides across the entire Pin1 sequence (Table S3). Peptide deuteration levels (% D) for each peptide at each time-point were determined using eq 1.

1 $$\%\;D=\left(m-m_{0}\right)/\left(m_{100}-m_{0}\right)\times 100$$

where *m* is the experimental peptide centroid mass, *m*_0_ is the nondeuterated peptide centroid mass, and *m*_100_ is the experimentally determined maximally deuterated peptide centroid mass.

The % *D* difference between Pin1 with and without CRMP2_507–517_ pT509, pT514 peptide was mapped onto the Pin1 structure (PDB: 1f8a) and visualized using Chimera v1.9.

## Supplementary Material



## Notes

Additional experimental details, data tables, and native MS and HDX-MS figures (PDF)
All MS data within this manuscript have been deposited to the ProteomeXchange Consortium via the PRIDE partner repository with the data set identifier PXD08272 and 10.6019/PXD078272
