# Supplementary material for: Mapping Phosphorylation-Specific Pin1–CRMP2 Interactions Using an Integrated Mass Spectrometry Approach
Source: ACS Chem Biol. 2026 May 26;21(6):1487–95. doi: 10.1021/acschembio.6c00227 (PMC13288452; doi:10.1021/acschembio.6c00227)
Supplement: Supplementary file 1 [file cb6c00227_si_001.pdf]

# Supplementary Information

# Mapping Phosphorylation-Specific Pin1-CRMP2 Interactions using an Integrated Mass Spectrometry Approach

Danielle F. Kay[a], Nikolas J. Brooks[a], Simon G. Caulton[a], Hiruni S. Jayasekera[a], Andrew L. Lovering[a], Aneika C. Leney[a] *

[a] School of Biosciences, University of Birmingham, Edgbaston, Birmingham, B15 2TT, UK

**Contents**


Figure S1. Native mass spectra of unphosphorylated CRMP2$_{507\text{-}517}$ binding to Pin1……………………………S2

Figure S2. Native mass spectra of CRMP2 pS27 (a), Cdc25 pT48 (b), Tau pS235 (c), and Tau pT231, pS235 (d) binding to Pin1……………………………………………………………………………………………S2

Figure S3. Native mass spectra of CRMP2$_{507\text{-}517}$ pT514 binding to Pin1…………………………………………S3

Figure S4. Peptide coverage of Pin1 protein during HDX-MS……………………………………………………S3

Figure S5. HDX heat map of apo Pin1 ……………………………………………………………………….S4

Figure S6. Deuterium uptake plots comparing apo Pin1, Pin1-CRMP2$_{507\text{-}517}$ and Pin1-CRMP2$_{507\text{-}517}$ pT509, pT514 complexes ….……………………………………………………...…………………………………S5-6

Figure S7. HDX difference profile on Pin1 comparing apo Pin1 and Pin1-CRMP2$_{507\text{-}517}$...................................S7

Figure S8. Structure of Pin1 WW domain interacting with a phosphopeptide…………………………………..S8

Figure S9. HDX difference profile on Pin1 comparing apo Pin1 and the Pin1-CRMP2$_{507\text{-}517}$ pT509, pT514 …………………………………………………………………………………………………………..S9

Figure S10. Deuterium uptake plots for peptides covering the PPIase binding site comparing apo Pin1, Pin1-CRMP2$_{507\text{-}517}$ and Pin1-CRMP2$_{507\text{-}517}$ pT509, pT514…………………………………………………………S10

Figure S11. Native MS of Pin1-CRMP2$_{507\text{-}517}$ pT509, pT514 complex under HDX-MS conditions.…………………………………………………………………………………………..S11

Figure S12. AlphaFold3 webserver predictions of the CRMP2-Pin1 interaction……………………………….S12

Figure S13. Image of home-built HDX-MS setup…………………………………………………………........S13

Table S1: Synthetic Peptide Sequences used to measure p27 CRMP2A, Cdc25 and Tau interactions with Pin1………………………………...…………………………………………………………......................S14

Table S2: Theoretical and measured masses of CRMP2 peptides and Pin1 complexes......................................S15

Table S3: HDX-MS sample information.............................................................................................................S16

Table S4: Raw HDX-MS data for apo Pin1, Pin1-CRMP2$_{507\text{-}517}$ and Pin1-CRMP2$_{507\text{-}517}$ pT509, pT514…………………………………………………………………………………………….......S17

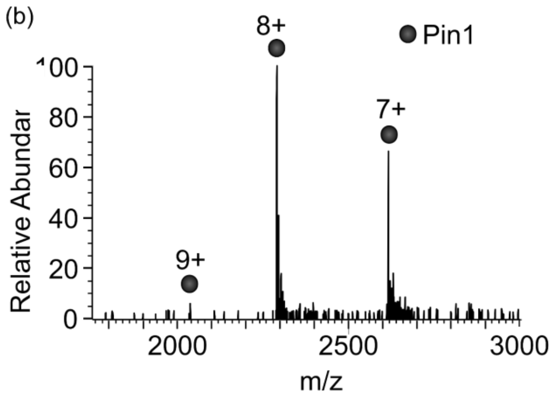


**Figure S1. Native mass spectra of Pin1 (5 µM) incubated with 20 µM unphosphorylated CRMP2$_{507\text{-}517}$.** Black circles represent peaks corresponding to apo Pin1. No complex formation was observed.

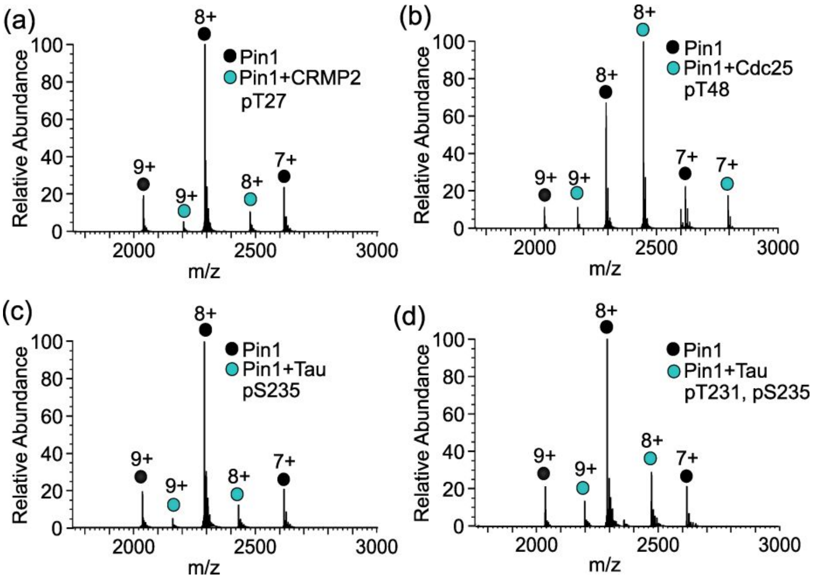


**Figure S2. Native mass spectra of Pin1 (5 µM) incubated with 20 µM CRMP2 pS27 (a), Cdc25 pT48 (b), Tau pS235 (c), Tau pT231, pS235 (d).** Black and blue circles represent peaks corresponding to apo Pin1 and the associated Pin1-peptide (1:1) complex, respectively.

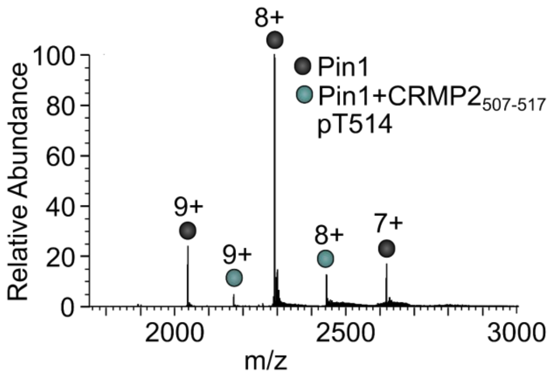


**Figure S3. Native mass spectra of Pin1 (5 µM) incubated with 20 µM CRMP2$_{507\text{-}517}$ pT514.** Black and teal circles represent peaks corresponding to apo Pin1 and the Pin1-CRMP2$_{507\text{-}517}$ pT514 complex, respectively.

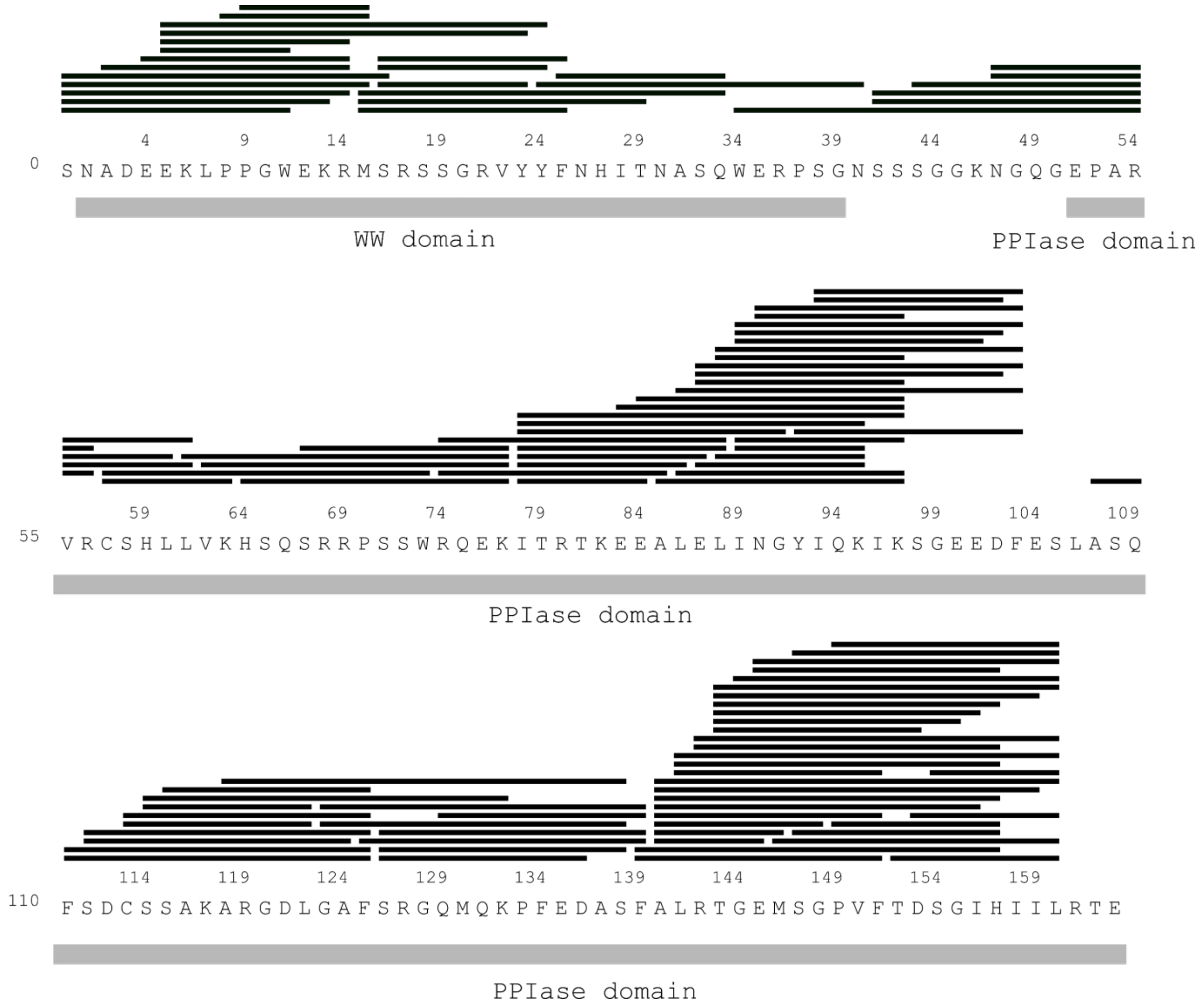


**Figure S4. Peptide coverage of Pin1 following online digestion.** All peptides identified are illustrated by black lines above the Pin1 sequence. The WW domain and PPIase domain are shown in grey boxes below the protein sequence. A total number of 113 peptides were analyzed for deuterium uptake providing a sequence coverage of 96%.

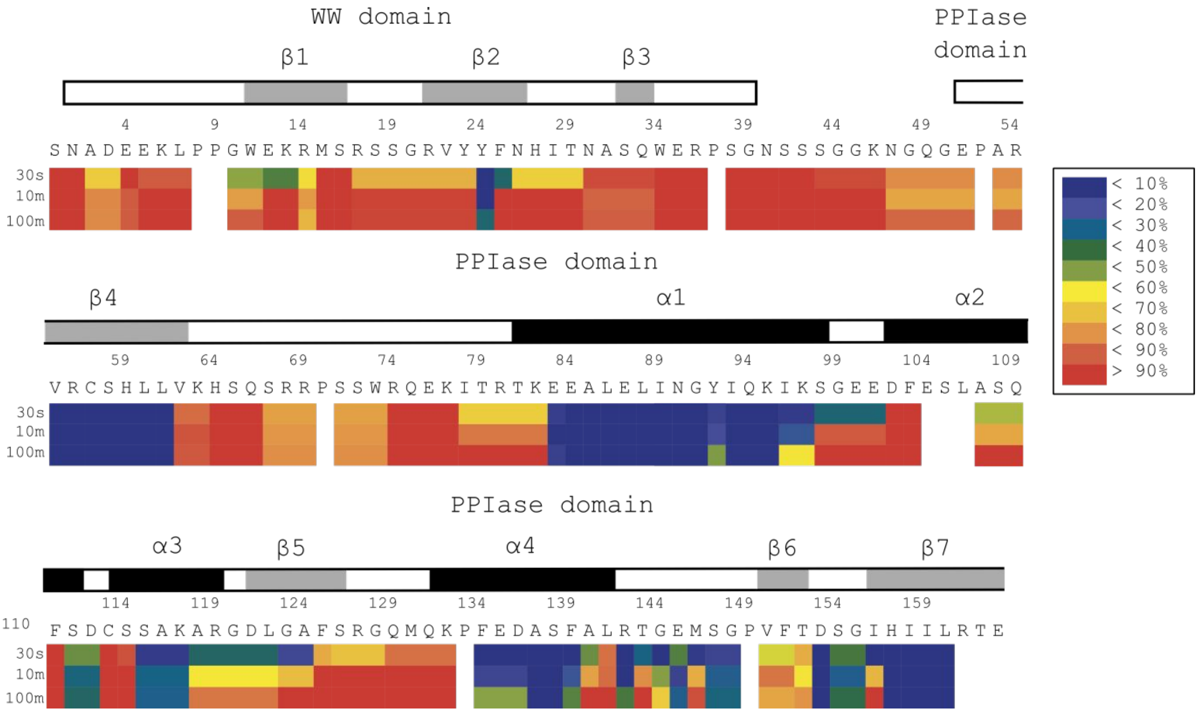


**Figure S5. HDX profile for apo Pin1.** Blue regions represent regions of low deuterium labelling (~10-30 %) and orange/red regions correspond to regions of high deuterium labelling ( > 80 %). Each line represents a different HDX time-point (30 seconds, 10 minutes and 100 minutes). The WW domain and PPIase domain are shown in black outline boxes above the protein sequence. β-sheets and α-helices are labelled within the boxes as grey and black, respectively.

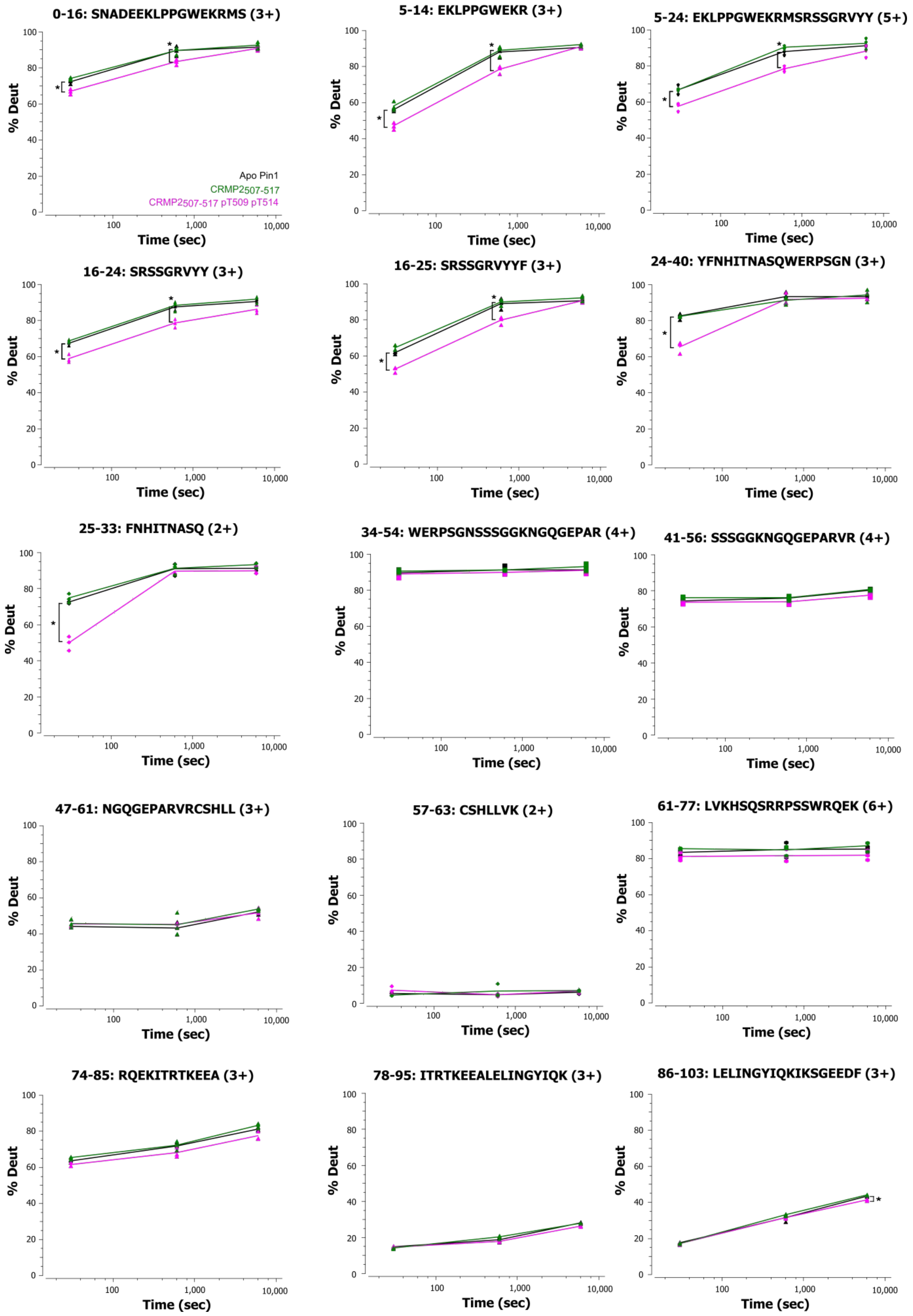
0-16: SNADEEKLPPGWEKRMS (3+)
5-14: EKLPPGWEKR (3+)
5-24: EKLPPGWEKRMSRSSGRVYY (5+)
16-24: SRSSGRVYY (3+)
16-25: SRSSGRVYYF (3+)
24-40: YFNHITNASQWERPSGN (3+)
25-33: FNHITNASQ (2+)
34-54: WERPSGNSSSGGKNGQGEPAR (4+)
41-56: SSSGGKNGQGEPARVR (4+)
47-61: NGQGEPARVRCSHLL (3+)
57-63: CSHLLVK (2+)
61-77: LVKHSQSRRPSSWRQEK (6+)
74-85: RQEKITRTKEEA (3+)
78-95: ITRTKEEALELINGYIQK (3+)
86-103: LELINGYIQKIKSGEEDF (3+)
% Deut
Time (sec)
Apo Pin1
CRMP2507-517
CRMP2507-517 pT509 pT514

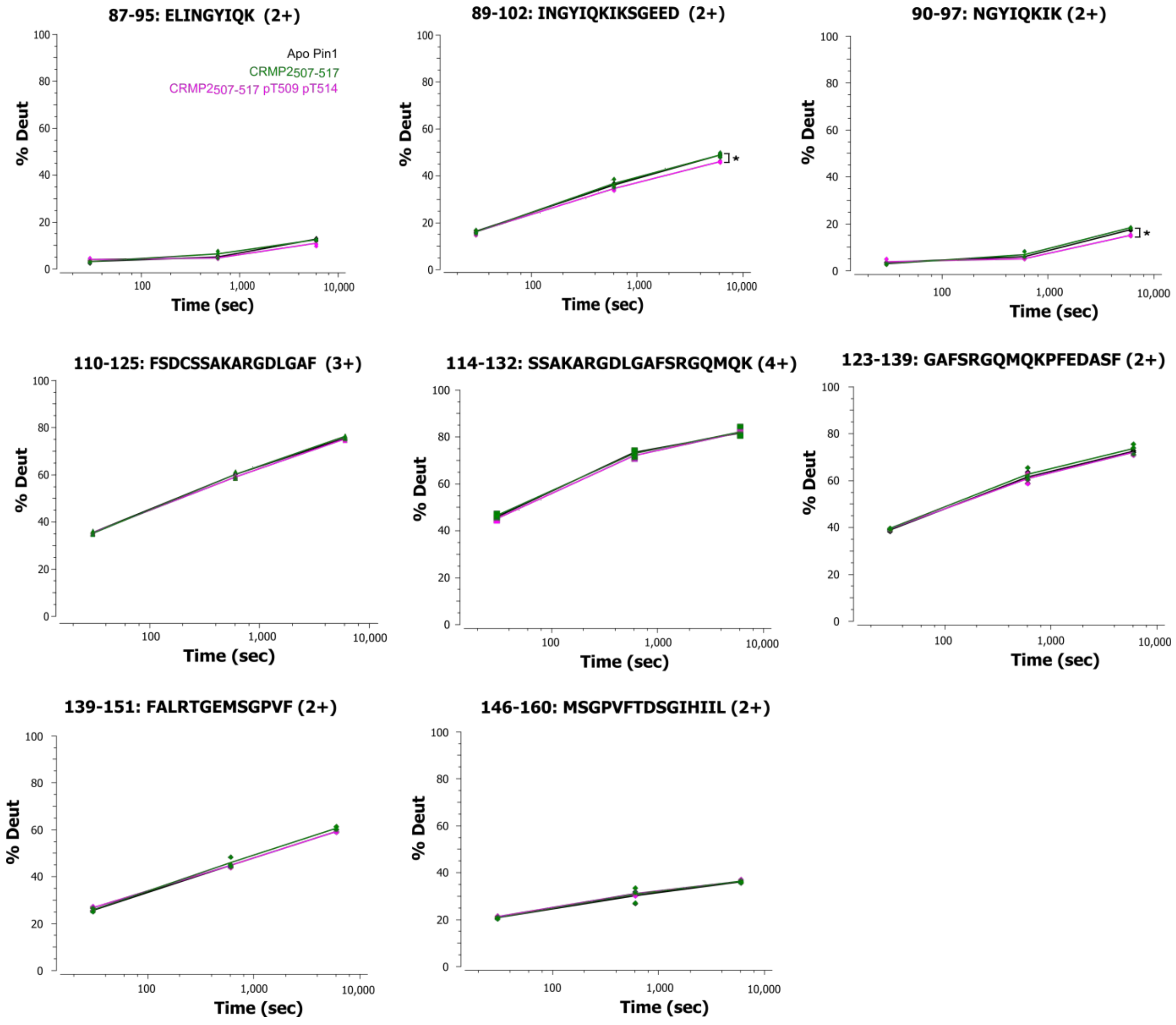


**Figure S6. Deuterium uptake over time for select peptides covering the Pin1 sequence.** % deuterium (% Deut) incorporation is shown at 30 seconds, 10 minutes and 100 minutes for apo Pin1 (black), Pin1-CRMP2$_{507\text{-}517}$ (green) and Pin1-CRMP2$_{507\text{-}517}$ pT509, pT514 (pink). These selected 23 peptides are spread across the Pin1 sequence. Peptide sequences and their residue numbers are shown above the plots with the corresponding charge state. * represents significant differences that were observed between the apo Pin1 and Pin1-bound states ($p \leq 0.05$).

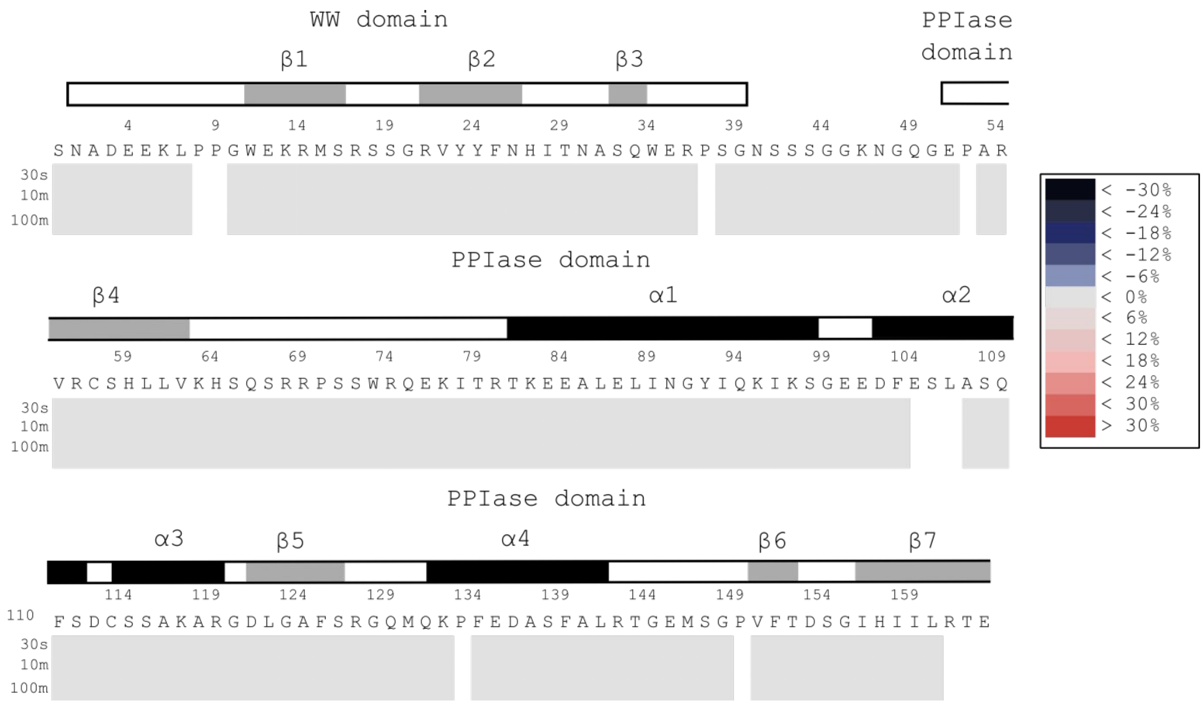


**Figure S7. HDX difference profile on the pseudo-residue level between apo Pin1 and Pin1 incubated with the unphosphorylated CRMP2$_{507\text{-}517}$ peptide.** Each line represents a different time-point (30 seconds, 10 minutes and 100 minutes). The WW domain and PPIase domain are shown in black outline boxes above the protein sequence. β-sheets and α-helices are labelled within the boxes as grey and black, respectively.

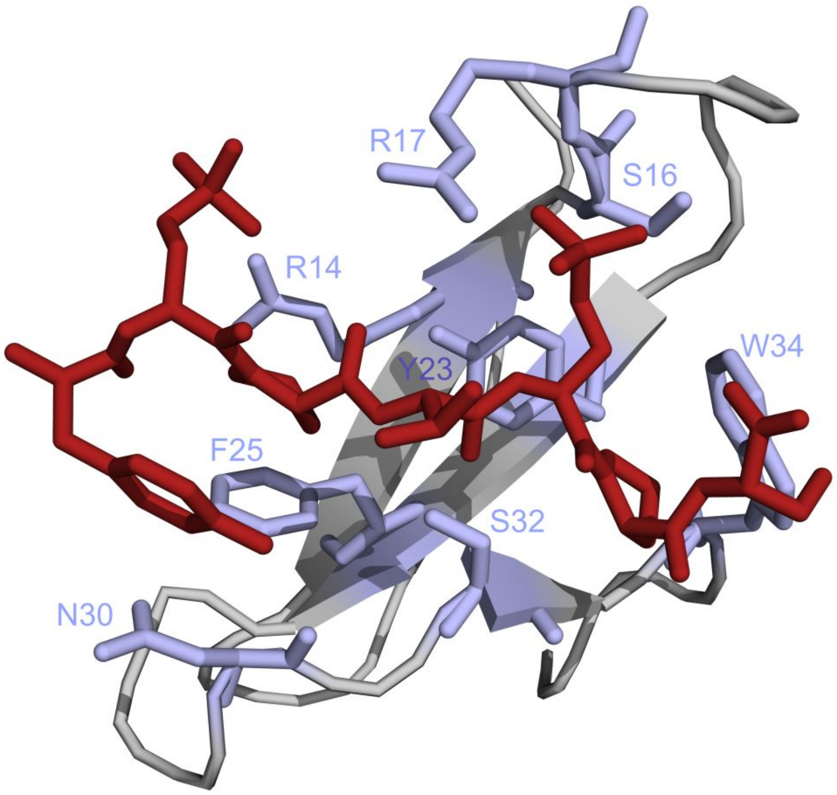


**Figure S8. The WW domain binding pocket.** Crystal structure of the Pin1 WW domain (grey) bound to doubly phosphorylated RNA polymerase peptide YpSPTpSPS (red) (pdb 1f8a). Key interacting residues on the WW domain are labelled and include Arg14, Ser16, Arg17, Tyr23, Phe25, Asn30, Ser32 and Trp34.

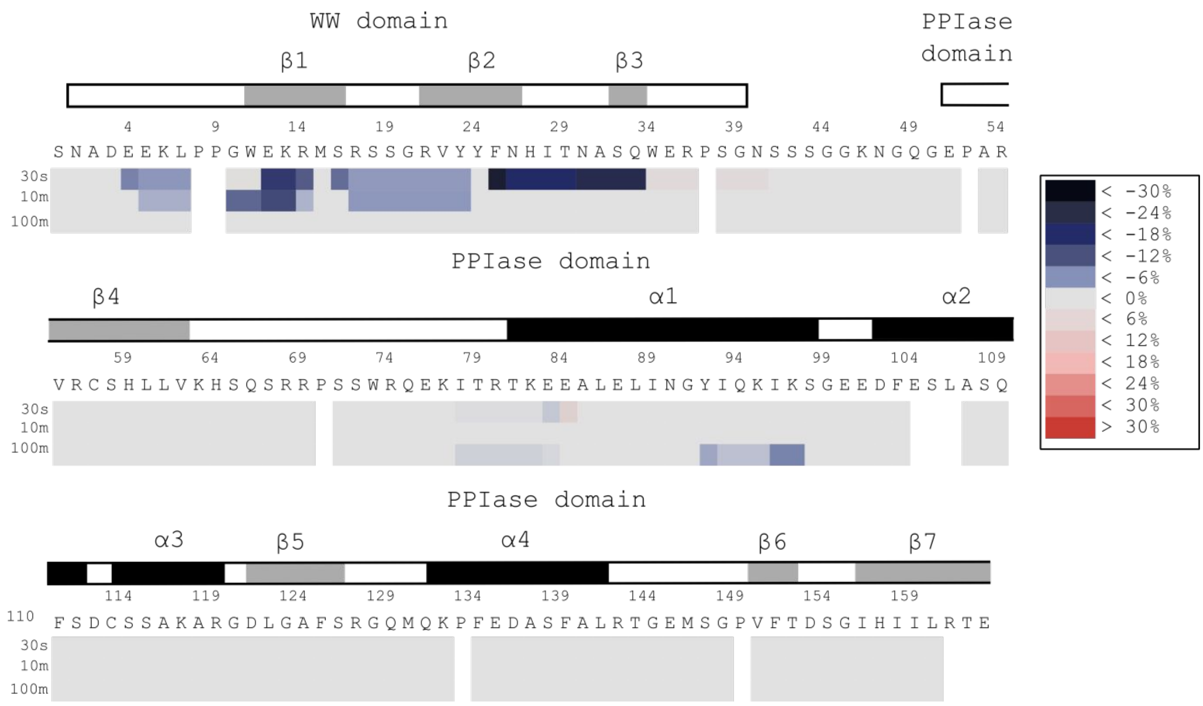


**Figure S9. HDX difference profile on the pseudo-residue level for Pin1 in the presence and absence of CRMP2$_{507\text{-}517}$ pT509, pT514.** Blue represents regions protected from deuterium exchange whilst red represents deprotected regions. Each line represents a different time-point (30 seconds, 10 minutes and 100 minutes). The WW domain and PPIase domain are shown in black outline boxes above the protein sequence. β-sheets and α-helices are labelled within the boxes as grey and black, respectively.

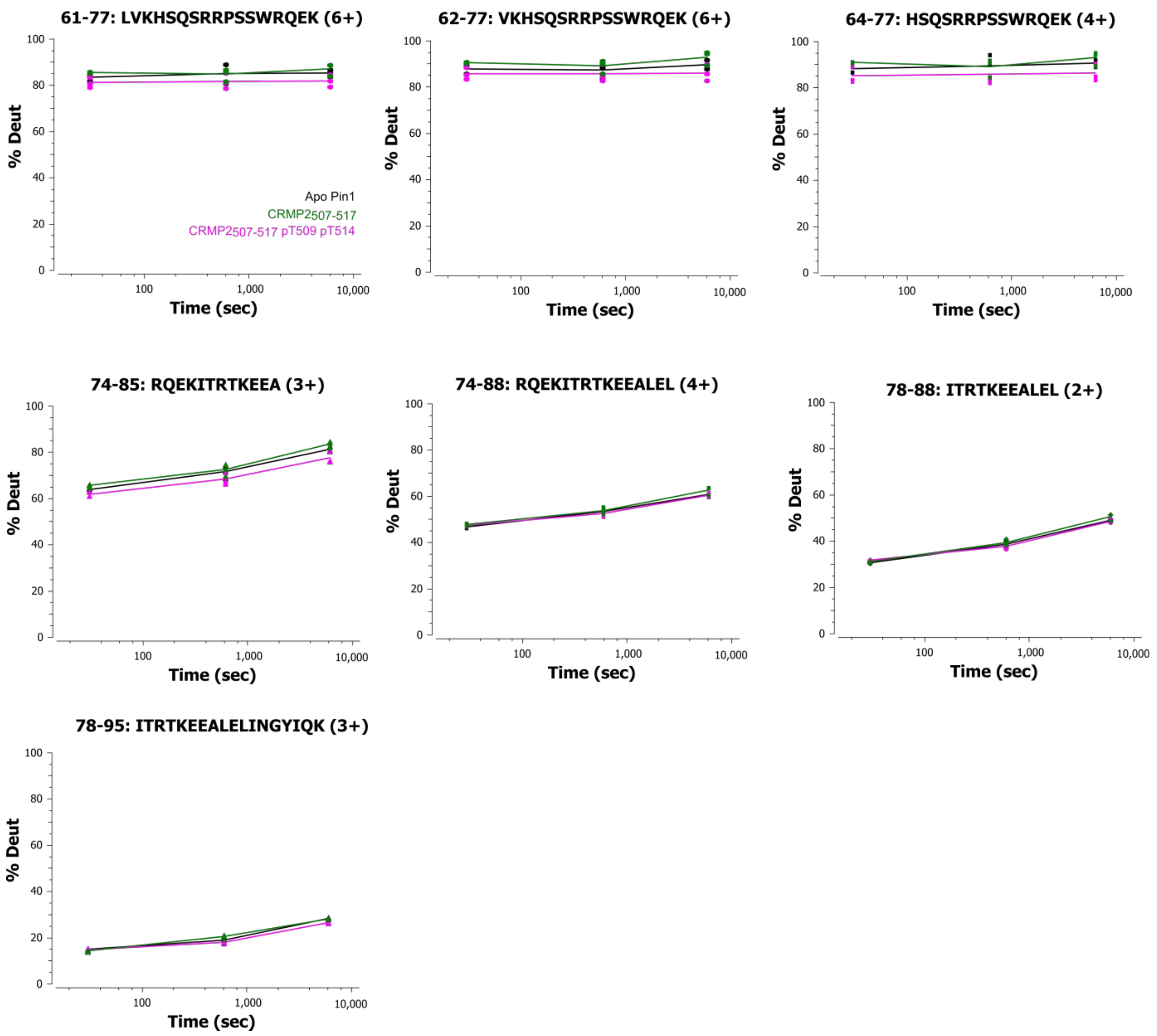


**Figure S10. Deuterium uptake over time for select peptides within the PPIase domain of Pin1.** % deuterium (% Deut) incorporation is shown at 30 seconds, 10 minutes and 100 minutes for apo Pin1 (black), Pin1-CRMP2$_{507-517}$ (green) and Pin1-CRMP2$_{507-517}$ pT509, pT514 (pink). Peptide sequences and their residue numbers are shown above the plots with the corresponding charge state. * represents significant differences that were observed between the apo Pin1 and Pin1-bound states ($p \leq 0.05$).

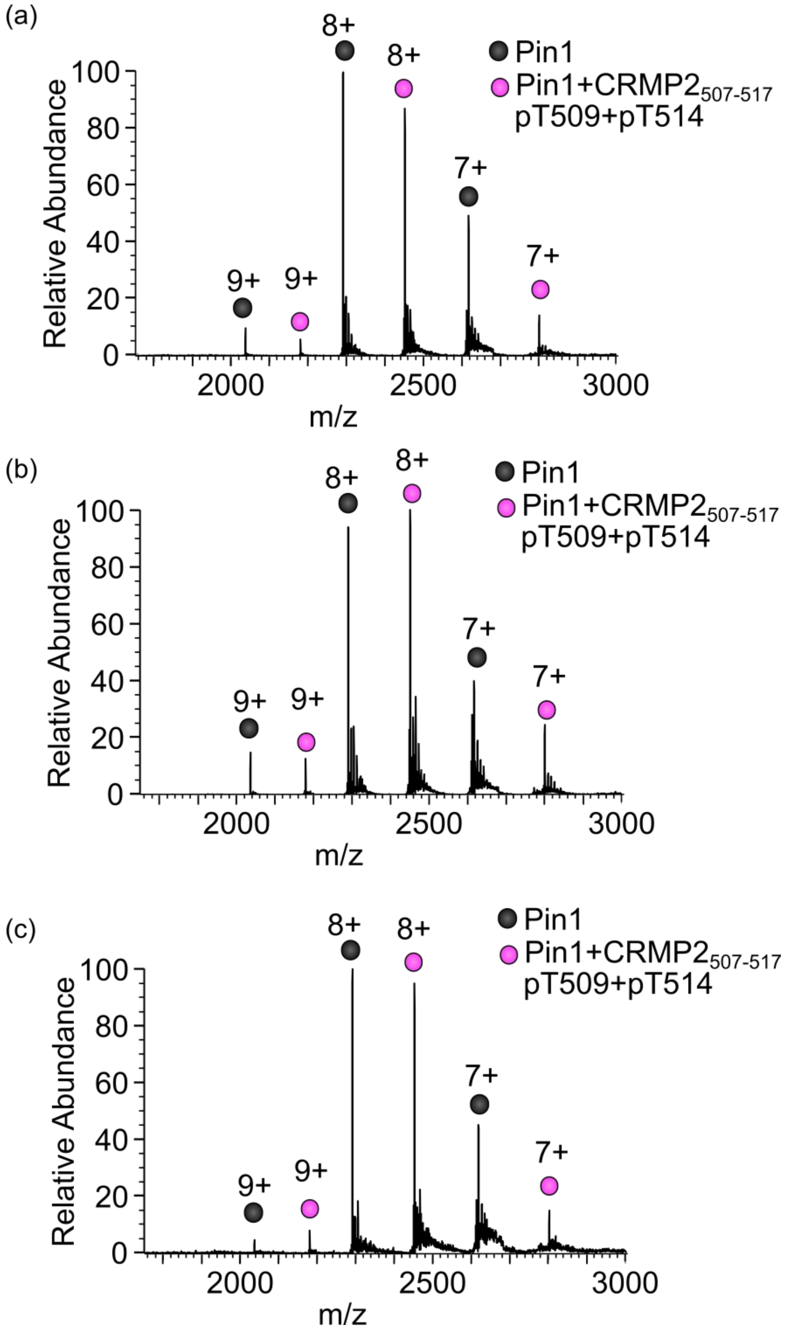


**Figure S11. Native mass spectra of Pin1-CRMP2$_{507\text{-}517}$ pT509 pT514 complex at same conditions as HDX-MS experiments showing the complex remains over the time course of the HDX reaction.** 5 μM Pin1 was incubated with 50 μM CRMP2$_{507\text{-}517}$ pT509 pT514 and the complex formed analysed by native MS after ~1 minute (a), 10 minutes (b) and 100 minutes (c). Black and pink dots represent peaks for apo Pin1 and the Pin1-CRMP2 complex, respectively.

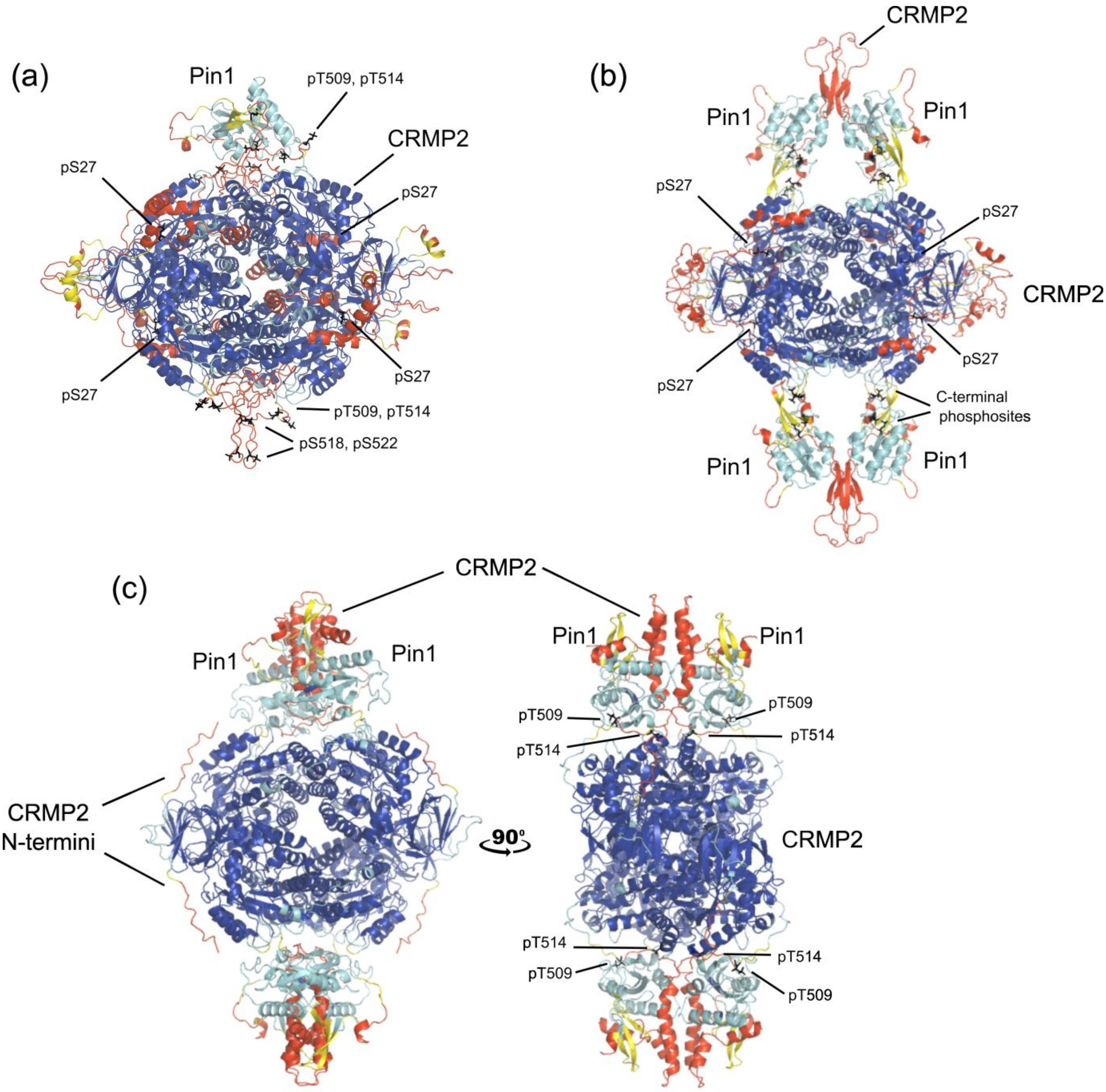


**Figure S12. AlphaFold3 webserver predictions of full length CRMP2 tetramer (Uniprot Accession ID: A0A1C7CYX9) in complex with one (a) or four (b, c) molecules of Pin1.** Four full length CRMP2 sequences were submitted to the AlphaFold3 webserver either with all the phosphorylation sites pSer27, pT509, pT514, pS518, pS522 (a, b) or only the pT509 and pT514 phosphosites on each molecule within the tetramer. Note, these phosphosites correspond to pSer27, pT614, pT619, pS623, pS627 within the full length CRMP2 sequence. The CRMP2 sequences were submitted either with one molecule of Pin1 (a) or four molecules of Pin1 (b,c). Phosphosites are shown as black sticks. The structures are colored according to their pIDDT scores; very high pIDDT >90 blue, confident (90 > plDDT > 70) cyan, low (70 > plDDT > 50) yellow, very low (plDDT < 50) red. Note, the CRMP2 C-terminal tails in all structures have very low pIDDT scores. In each case, Pin1 is located in a different orientation to the CRMP2 tetramer.

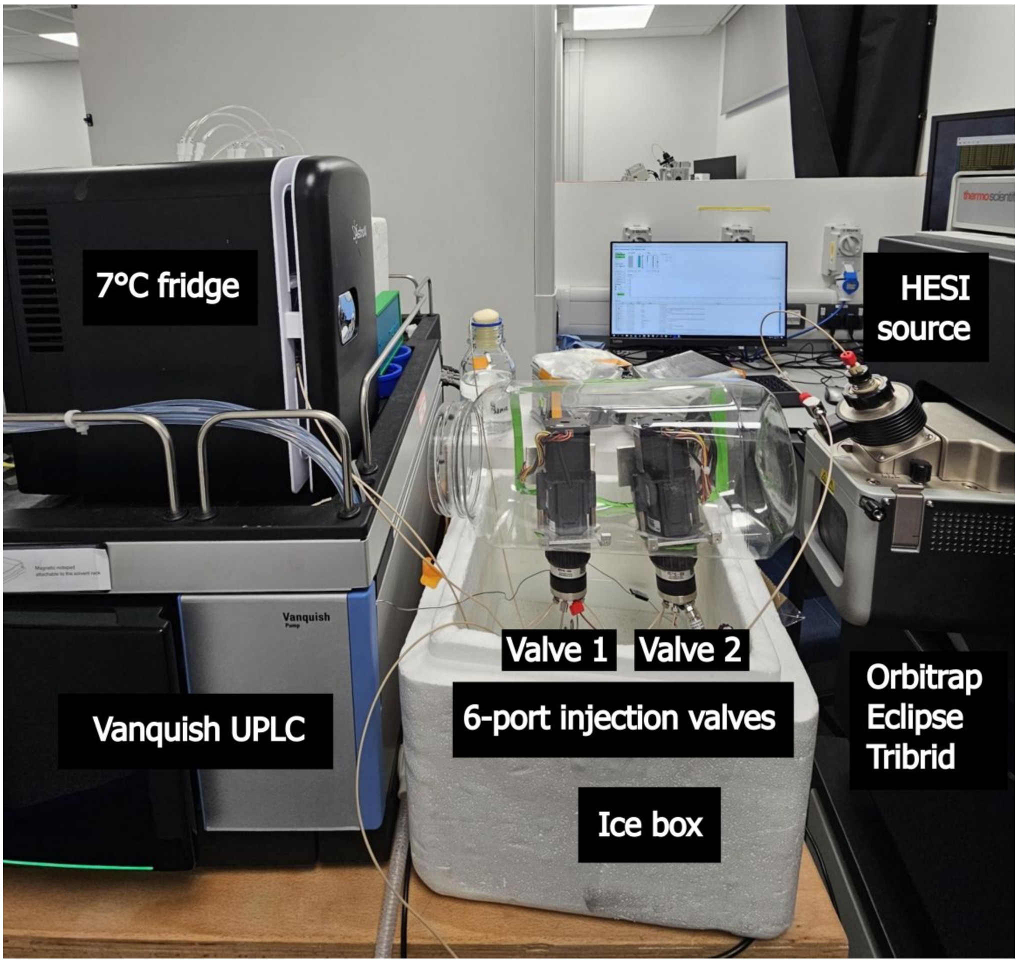


**Figure S13. Home-built HDX-MS set-up. Protein** sample is injected into 6 port valve (valve 1). Valve 1 is then switched, and using an MX-Class Auxiliary Pump (located on a shelf underneath the Vanquish UPLC), the protein sample transferred onto the dual nepenthesin-2/pepsin POROS column (kept at 7 °C in the mini fridge) at a flow rate of 0.1 ml/min. The resulting peptides are collected immediately on a C18 trap column (kept at 0 °C in the ice-box) that is connected to another 6 port valve (valve 2). After the peptides on trap column are desalted, valve 2 is switched and the peptides transferred to a C18 analytical column (kept at 0 °C in the ice-box). Peptides are then separated using a solvent gradient provided by the Vanquish UPLC and the eluted peptides directly infused into the Orbitrap Eclipse Tribrid mass spectrometer via use of the HESI source. Note that all mobile phases were kept in an ice bath throughout.

**Table S1. Synthetic peptide sequences for determining Pin1 interactions with the phosphorylated sites on the N-terminus of CRMP2A, CdcC25 and Tau.**

| **Peptide name** | **Peptide sequence** |
|---|---|
| $CRMP2_{21\text{-}33}$ pS27 | $_{21}$CNLGSG**S**PKPRQK$_{33}$ |
| $Cdc25C_{44\text{-}53}$ pT48 | $_{44}$EQPL**T**PVTDL$_{53}$ |
| $Tau_{231\text{-}240}$ pS235 | $_{231}$TPPK**S**PSSAK$_{240}$ |
| $Tau_{229\text{-}240}$ pT231, pS235 | $_{229}$VR**T**PPK**S**PSSAK$_{240}$ |

**Table S2. Theoretical and measured masses of CRMP2 peptides and Pin1 complexes used within this study**. N/A indicates no complex was detected. All measured masses has a standard deviation of < 0.6 Da.

| **Peptide/Protein Complex** | **Theoretical Mass (Da)** | **Measured Mass (Da)** |
|---|---|---|
| $CRMP2_{507-525}$ | 1857.0 | 1857.0 |
| $CRMP2_{507-525}$ pS522 | 1937.0 | 1937.0 |
| $CRMP2_{507-525}$ pS518, pS522 | 2017.0 | 2017.0 |
| $CRMP2_{507-525}$ pT514, pS518, pS522 | 2097.0 | 2069.9 |
| $CRMP2_{507-525}$ pT509, pT514, pS518, pS522 | 2177.0 | 2176.9 |
| $CRMP2_{512-520}$ pT514 | 981.5 | 981.5 |
| $CRMP2_{512-520}$ pT514, pS518 | 1061.5 | 1061.4 |
| $CRMP2_{507-517}$ | 1127.6 | 1127.6 |
| $CRMP2_{507-517}$ pT514 | 1207.6 | 1207.6 |
| $CRMP2_{507-517}$ pT509 | 1207.6 | 1207.6 |
| $CRMP2_{507-517}$ pT509, pT514 | 1287.6 | 1287.6 |
| $CRMP2_{21-33}$ pS27 | 1491.7 | 1491.7 |
| $Cdc25C_{44-53}$ pT48 | 1232.6 | 1232.6 |
| $Tau_{231-240}$ pS235 | 1119.6 | 1119.5 |
| $Tau_{229-240}$ pT231, pS235 | 1454.7 | 1454.7 |
| Pin1 | 18313.3 | 18313.3 |
| Pin1-$CRMP2_{507-525}$ | 20170.3 | N/A |
| Pin1-$CRMP2_{507-525}$ pS522 | 20250.3 | 20250.3 |
| Pin1-$CRMP2_{507-525}$ pS518, pS522 | 20330.0 | 20330.3 |
| Pin1-$CRMP2_{507-525}$ pT514, pS518, pS522 | 20410.3 | 20410.5 |
| Pin1-$CRMP2_{507-525}$ pT509, pT514, pS518, pS522 | 20490.3 | 20490.0 |
| Pin1-$CRMP2_{512-520}$ pT514 | 19294.8 | 19294.4 |
| Pin1-$CRMP2_{512-520}$ pT514, pS518 | 19374.8 | 19374.6 |
| Pin1-$CRMP2_{507-517}$ | 19440.9 | N/A |
| Pin1-$CRMP2_{507-517}$ pT514 | 19520.9 | 19520.9 |
| Pin1-$CRMP2_{507-517}$ pT509 | 19520.9 | 19521.0 |
| Pin1-$CRMP2_{507-517}$ pT509, pT514 | 19600.9 | 19600.4 |
| $CRMP2_{21-33}$ pS27 | 19805.0 | 19804.4 |
| Pin1-$Cdc25C_{44-53}$ pT48 | 19545.9 | 19544.8 |
| Pin1-$Tau_{231-240}$ pS235 | 19432.9 | 19432.2 |
| Pin1-$Tau_{229-240}$ pT231, pS235 | 19768.0 | 19767.7 |

**Table S3. HDX-MS sample information**

| Dataset | Apo Pin1 | Pin1-CRMP2$_{517\text{-}525}$ pS509, pS514 | Pin1-CRMP2$_{517\text{-}525}$ |
|---|---|---|---|
| HDX reaction details | Final $D_2O$ concentration = 90 % $D_2O$ in 50 mM ammonium acetate<br>$P_D$ = 7.2<br>Temperature = room temperature | | |
| HDX time course | 30 seconds, 10 minutes and 100 minutes | | |
| HDX controls | Maximally-labelled apo Pin1 control | | |
| Back-exchange (mean and range) | 31.6 % ( 20.7 % – 47.7 %) | | |
| Number of peptides | 113 | | |
| Sequence coverage | 96.3% | | |
| Average peptide redundancy | 9.3 | | |
| Technical replicates | 3 | 3 | 3 |
| Significant differences in HDX | 95% confidence | | |

**Table S4. Raw HDX-MS data for apo Pin1, Pin1-CRMP2$_{507-517}$ and Pin1-CRMP2$_{507-517}$ pT509 pT514.** The values presented are averages across three replicates.

| | | | | | Uptake - Apo Pin1 (average D) | | | Uptake - Pin1 + CRMP2_507-517 pT509 pT514 (D) | | | Uptake - Pin1 + CRMP2_507-517 (D) | | | Uptake - Control Sample (D) | Uptake error (SD) Apo Pin1 | | | Uptake error (SD) Pin1 + CRMP2_507-517 pT509 pT514 (D) | | | Uptake error (SD) Pin1 + CRMP2_507-517 (D) | | | Uptake error (SD) - Control Sample (D) |
|---|---|---|---|---|---|---|---|---|---|---|---|---|---|---|---|---|---|---|---|---|---|---|---|---|
| Start | End | Sequence | Charge | Retention time (min) | 30 s | 10 min | 100 min | 30 s | 10 min | 100 min | 30 s | 10 min | 100 min | MAX D20 | 30 s | 10 min | 100 min | 30 s | 10 min | 100 min | 30 s | 10 min | 100 min | MAX D20 |
| 0 | 11 | SNADEEKLPPGW | 2 | 7.2 | 5.44 | 6.06 | 6.26 | 5.44 | 5.95 | 6.31 | 5.54 | 6.06 | 6.34 | 6.95 | 0.06 | 0.23 | 0.04 | 0.12 | 0.12 | 0.16 | 0.04 | 0.11 | 0.12 | 0.07 |
| 0 | 13 | SNADEEKLPPGWEK | 3 | 6.28 | 6.45 | 7.94 | 8.16 | 6.21 | 7.61 | 8.32 | 6.69 | 8.00 | 8.28 | 8.95 | 0.07 | 0.27 | 0.08 | 0.10 | 0.16 | 0.12 | 0.09 | 0.19 | 0.13 | 0.11 |
| 0 | 14 | SNADEEKLPPGWEKR | 3 | 5.97 | 7.20 | 9.27 | 9.42 | 6.71 | 8.74 | 9.55 | 7.43 | 9.17 | 9.58 | 10.38 | 0.09 | 0.27 | 0.08 | 0.11 | 0.15 | 0.14 | 0.09 | 0.24 | 0.18 | 0.10 |
| 0 | 15 | SNADEEKLPPGWEKRM | 3 | 6.5 | 7.76 | 9.88 | 10.06 | 7.25 | 9.26 | 10.12 | 7.93 | 9.83 | 10.14 | 11.00 | 0.08 | 0.21 | 0.08 | 0.15 | 0.17 | 0.19 | 0.06 | 0.11 | 0.19 | 0.12 |
| 0 | 16 | SNADEEKLPPGWEKRMS | 3 | 6.3 | 8.59 | 10.65 | 10.88 | 8.09 | 10.09 | 10.99 | 8.75 | 10.60 | 10.95 | 11.89 | 0.09 | 0.27 | 0.08 | 0.15 | 0.16 | 0.16 | 0.06 | 0.19 | 0.20 | 0.12 |
| 2 | 14 | ADEEKLPPGWEKR | 3 | 6 | 6.25 | 8.09 | 8.41 | 5.78 | 7.80 | 8.54 | 6.42 | 8.39 | 8.61 | 8.99 | 0.09 | 0.19 | 0.23 | 0.06 | 0.20 | 0.10 | 0.11 | 0.02 | 0.23 | 0.34 |
| 4 | 14 | EEKLPPGWEKR | 3 | 5.81 | 4.50 | 6.40 | 6.60 | 3.85 | 5.89 | 6.62 | 4.51 | 6.44 | 6.70 | 7.24 | 0.05 | 0.12 | 0.05 | 0.12 | 0.16 | 0.05 | 0.12 | 0.14 | 0.07 | 0.09 |
| 5 | 11 | EKLPPGW | 2 | 7.06 | 2.02 | 2.69 | 2.83 | 1.87 | 2.41 | 2.73 | 1.92 | 2.58 | 2.70 | 2.99 | 0.02 | 0.03 | 0.02 | 0.04 | 0.03 | 0.05 | 0.02 | 0.01 | 0.03 | 0.07 |
| 5 | 14 | EKLPPGWEKR | 3 | 5.67 | 3.48 | 5.47 | 5.62 | 2.96 | 4.94 | 5.73 | 3.57 | 5.47 | 5.67 | 6.20 | 0.04 | 0.14 | 0.02 | 0.10 | 0.11 | 0.06 | 0.12 | 0.14 | 0.04 | 0.05 |
| 5 | 23 | EKLPPGWEKRMSRSSGRVY | 5 | 5.6 | 9.54 | 12.29 | 12.53 | 8.66 | 11.44 | 12.57 | 9.71 | 12.26 | 12.58 | 13.64 | 0.12 | 0.21 | 0.09 | 0.28 | 0.29 | 0.22 | 0.08 | 0.05 | 0.26 | 0.15 |
| 5 | 24 | EKLPPGWEKRMSRSSGRVYY | 5 | 6.21 | 9.66 | 12.71 | 13.19 | 8.40 | 11.45 | 12.88 | 9.58 | 12.96 | 13.27 | 14.44 | 0.30 | 0.23 | 0.04 | 0.28 | 0.18 | 0.39 | 0.06 | 0.10 | 0.37 | 0.07 |
| 8 | 15 | PPGWEKRM | 2 | 6.2 | 2.91 | 4.53 | 4.64 | 2.52 | 4.00 | 4.55 | 2.95 | 4.40 | 4.53 | 5.15 | 0.07 | 0.09 | 0.01 | 0.04 | 0.11 | 0.06 | 0.07 | 0.10 | 0.15 | 0.12 |
| 9 | 15 | PGWEKRM | 2 | 6.18 | 3.09 | 4.75 | 4.92 | 2.62 | 4.31 | 5.01 | 3.16 | 4.82 | 4.96 | 5.36 | 0.03 | 0.11 | 0.04 | 0.05 | 0.14 | 0.06 | 0.09 | 0.11 | 0.05 | 0.06 |
| 15 | 25 | MSRSSGRVYYF | 3 | 7.04 | 4.80 | 6.74 | 6.82 | 4.29 | 6.23 | 7.01 | 4.95 | 6.66 | 6.90 | 7.52 | 0.10 | 0.22 | 0.07 | 0.11 | 0.18 | 0.12 | 0.07 | 0.08 | 0.16 | 0.15 |
| 15 | 29 | MSRSSGRVYYFNHIT | 4 | 6.49 | 6.29 | 8.98 | 9.02 | 5.19 | 8.35 | 9.26 | 6.58 | 8.94 | 9.23 | 9.89 | 0.12 | 0.29 | 0.08 | 0.23 | 0.13 | 0.20 | 0.12 | 0.08 | 0.28 | 0.15 |
| 15 | 33 | MSRSSGRVYYFNHITNASQ | 4 | 6.37 | 9.51 | 12.77 | 12.67 | 7.30 | 12.09 | 12.81 | 9.82 | 12.64 | 12.80 | 13.76 | 0.10 | 0.27 | 0.08 | 0.43 | 0.10 | 0.33 | 0.21 | 0.14 | 0.32 | 0.19 |
| 16 | 23 | SRSSGRVY | 3 | 5.11 | 4.50 | 5.38 | 5.36 | 4.18 | 5.02 | 5.33 | 4.61 | 5.34 | 5.38 | 6.00 | 0.04 | 0.05 | 0.05 | 0.05 | 0.10 | 0.07 | 0.04 | 0.03 | 0.06 | 0.04 |
| 16 | 24 | SRSSGRVYY | 3 | 5.69 | 4.51 | 5.86 | 6.06 | 4.14 | 5.52 | 6.06 | 4.53 | 5.83 | 6.05 | 6.68 | 0.05 | 0.10 | 0.07 | 0.13 | 0.13 | 0.14 | 0.04 | 0.15 | 0.10 | 0.08 |
| 16 | 25 | SRSSGRVYYF | 3 | 6.94 | 4.28 | 6.16 | 6.26 | 3.73 | 5.67 | 6.43 | 4.40 | 6.14 | 6.30 | 6.92 | 0.04 | 0.17 | 0.05 | 0.09 | 0.12 | 0.09 | 0.08 | 0.11 | 0.09 | 0.08 |
| 24 | 40 | YFNHITNASQWERPSGN | 3 | 6.67 | 9.95 | 11.24 | 11.25 | 7.98 | 11.18 | 11.28 | 9.96 | 11.04 | 11.38 | 12.06 | 0.19 | 0.29 | 0.12 | 0.33 | 0.33 | 0.25 | 0.04 | 0.21 | 0.35 | 0.17 |
| 25 | 33 | FNHITNASQ | 2 | 5.34 | 4.72 | 5.92 | 5.93 | 3.32 | 5.97 | 6.00 | 4.81 | 5.87 | 6.01 | 6.51 | 0.04 | 0.18 | 0.05 | 0.21 | 0.12 | 0.12 | 0.13 | 0.16 | 0.08 | 0.04 |
| 34 | 54 | WERPSGNSSSGGKNGQGEPAR | 4 | 5.2 | 15.00 | 15.26 | 15.28 | 15.13 | 15.30 | 15.49 | 14.85 | 14.97 | 15.26 | 16.74 | 0.09 | 0.33 | 0.33 | 0.31 | 0.13 | 0.23 | 0.13 | 0.04 | 0.27 | 0.13 |
| 41 | 56 | SSSGGKNGQGEPARVR | 4 | 4.85 | 9.36 | 9.56 | 10.11 | 9.49 | 9.53 | 10.01 | 9.58 | 9.57 | 10.15 | 12.59 | 0.07 | 0.05 | 0.14 | 0.12 | 0.17 | 0.18 | 0.05 | 0.16 | 0.05 | 0.09 |
| 41 | 61 | SSSGGKNGQGEPARVRCSHLL | 3 | 5.87 | 7.78 | 7.52 | 8.43 | 8.17 | 7.88 | 8.80 | 7.80 | 7.99 | 8.69 | 13.25 | 0.11 | 0.40 | 0.15 | 0.16 | 0.12 | 0.16 | 0.06 | 0.28 | 0.29 | 0.20 |
| 43 | 60 | SGGKNGQGEPARVRCSHL | 4 | 5.53 | 7.75 | 7.78 | 8.40 | 8.25 | 8.06 | 8.85 | 7.76 | 7.70 | 8.67 | 12.40 | 0.06 | 0.48 | 0.15 | 0.26 | 0.30 | 0.23 | 0.07 | 0.60 | 0.27 | 0.20 |
| 47 | 56 | NGQGEPARVR | 3 | 4.92 | 5.41 | 5.51 | 5.86 | 5.38 | 5.29 | 5.60 | 5.37 | 5.49 | 5.82 | 7.94 | 0.05 | 0.10 | 0.09 | 0.11 | 0.10 | 0.14 | 0.12 | 0.20 | 0.18 | 0.12 |
| 47 | 61 | NGQGEPARVRCSHLL | 3 | 6.15 | 4.29 | 4.20 | 5.07 | 4.50 | 4.45 | 5.09 | 4.35 | 4.30 | 5.10 | 9.72 | 0.04 | 0.27 | 0.14 | 0.16 | 0.09 | 0.25 | 0.18 | 0.48 | 0.07 | 0.10 |
| 57 | 63 | CSHLLVK | 2 | 5.56 | 0.22 | 0.20 | 0.25 | 0.30 | 0.20 | 0.29 | 0.18 | 0.27 | 0.29 | 4.06 | 0.02 | 0.01 | 0.04 | 0.07 | 0.02 | 0.04 | 0.00 | 0.12 | 0.02 | 0.02 |
| 57 | 73 | CSHLLVKHSQSRRPSSW | 4 | 5.48 | 5.23 | 5.44 | 5.45 | 5.49 | 5.63 | 5.76 | 5.33 | 5.38 | 5.61 | 9.36 | 0.07 | 0.20 | 0.07 | 0.20 | 0.14 | 0.16 | 0.03 | 0.04 | 0.20 | 0.19 |
| 61 | 77 | LVKHSQSRRPSSWRQEK | 6 | 4.89 | 8.78 | 8.94 | 8.97 | 9.29 | 9.36 | 9.38 | 8.93 | 8.86 | 9.10 | 10.52 | 0.12 | 0.35 | 0.13 | 0.21 | 0.31 | 0.24 | 0.02 | 0.23 | 0.24 | 0.21 |
| 62 | 77 | VKHSQSRRPSSWRQEK | 6 | 4.87 | 8.68 | 8.63 | 8.86 | 9.22 | 9.21 | 9.25 | 8.76 | 8.62 | 8.99 | 9.91 | 0.15 | 0.27 | 0.16 | 0.22 | 0.32 | 0.31 | 0.02 | 0.23 | 0.24 | 0.14 |
| 64 | 77 | HSQSRRPSSWRQEK | 4 | 4.93 | 8.50 | 8.61 | 8.74 | 8.83 | 8.91 | 8.95 | 8.65 | 8.48 | 8.85 | 9.66 | 0.11 | 0.40 | 0.12 | 0.31 | 0.41 | 0.31 | 0.02 | 0.30 | 0.24 | 0.10 |
| 67 | 77 | SRRPSSWRQEK | 4 | 4.99 | 6.72 | 6.66 | 6.81 | 6.96 | 6.98 | 7.00 | 6.91 | 6.89 | 6.95 | 7.61 | 0.09 | 0.43 | 0.13 | 0.14 | 0.26 | 0.20 | 0.03 | 0.01 | 0.13 | 0.11 |
| 74 | 85 | RQEKITRTKEEA | 3 | 4.85 | 6.01 | 6.73 | 7.65 | 6.13 | 6.79 | 7.70 | 6.15 | 6.79 | 7.84 | 9.44 | 0.06 | 0.21 | 0.09 | 0.09 | 0.22 | 0.20 | 0.05 | 0.19 | 0.10 | 0.13 |
| 74 | 88 | RQEKITRTKEEALEL | 4 | 6.08 | 5.39 | 6.16 | 7.00 | 5.58 | 6.19 | 7.10 | 5.50 | 6.19 | 7.20 | 11.52 | 0.03 | 0.19 | 0.08 | 0.07 | 0.14 | 0.09 | 0.04 | 0.13 | 0.17 | 0.10 |
| 78 | 84 | ITRTKEE | 2 | 4.61 | 2.94 | 3.56 | 4.48 | 2.97 | 3.49 | 4.40 | 3.01 | 3.56 | 4.57 | 5.07 | 0.01 | 0.22 | 0.02 | 0.02 | 0.04 | 0.04 | 0.02 | 0.24 | 0.06 | 0.03 |
| 78 | 86 | ITRTKEEAL | 3 | 5.49 | 2.86 | 3.55 | 4.44 | 3.00 | 3.51 | 4.46 | 2.89 | 3.55 | 4.53 | 6.95 | 0.01 | 0.13 | 0.05 | 0.07 | 0.13 | 0.14 | 0.02 | 0.14 | 0.08 | 0.06 |
| 78 | 87 | ITRTKEEALE | 3 | 5.32 | 2.65 | 3.40 | 4.26 | 2.86 | 3.37 | 4.33 | 2.69 | 3.41 | 4.39 | 7.64 | 0.01 | 0.13 | 0.06 | 0.08 | 0.15 | 0.16 | 0.02 | 0.12 | 0.11 | 0.07 |
| 78 | 88 | ITRTKEEALEL | 2 | 6.6 | 2.54 | 3.21 | 4.05 | 2.70 | 3.20 | 4.13 | 2.57 | 3.25 | 4.18 | 8.30 | 0.02 | 0.10 | 0.05 | 0.02 | 0.08 | 0.07 | 0.02 | 0.09 | 0.11 | 0.08 |

| | | | | | | | | | | | | | | | | | | | | | | | | |
|---|---|---|---|---|---|---|---|---|---|---|---|---|---|---|---|---|---|---|---|---|---|---|---|---|
| 78 | 91 | ITRTKEEALELING | 2 | 6.72 | 2.36 | 2.80 | 4.05 | 2.29 | 2.77 | 3.87 | 2.28 | 3.00 | 4.16 | 10.62 | 0.06 | 0.24 | 0.04 | 0.08 | 0.08 | 0.09 | 0.04 | 0.05 | 0.04 | 0.11 |
| 78 | 95 | ITRTKEEALELINGYIQK | 3 | 7.21 | 2.21 | 2.80 | 4.22 | 2.26 | 2.73 | 4.03 | 2.14 | 3.10 | 4.25 | 14.97 | 0.05 | 0.17 | 0.06 | 0.07 | 0.04 | 0.03 | 0.05 | 0.13 | 0.02 | 0.08 |
| 78 | 97 | ITRTKEEALELINGYIQKIK | 4 | 7.74 | 2.13 | 2.81 | 4.77 | 2.17 | 2.76 | 4.44 | 2.07 | 3.11 | 4.83 | 16.89 | 0.06 | 0.15 | 0.01 | 0.08 | 0.09 | 0.04 | 0.02 | 0.16 | 0.04 | 0.06 |
| 83 | 97 | EEALELINGYIQKIK | 3 | 8.41 | 0.29 | 0.39 | 1.50 | 0.38 | 0.44 | 1.21 | 0.29 | 0.65 | 1.61 | 13.12 | 0.06 | 0.13 | 0.05 | 0.11 | 0.04 | 0.14 | 0.04 | 0.11 | 0.05 | 0.12 |
| 84 | 97 | EALELINGYIQKIK | 3 | 7.85 | 0.19 | 0.49 | 1.46 | 0.33 | 0.33 | 1.18 | 0.20 | 0.43 | 1.52 | 12.34 | 0.06 | 0.00 | 0.02 | 0.13 | 0.11 | 0.03 | 0.11 | 0.20 | 0.08 | 0.02 |
| 85 | 97 | ALELINGYIQKIK | 3 | 7.54 | 0.24 | 0.47 | 1.57 | 0.29 | 0.41 | 1.24 | 0.20 | 0.52 | 1.51 | 10.67 | 0.03 | 0.03 | 0.05 | 0.07 | 0.04 | 0.02 | 0.00 | 0.10 | 0.03 | 0.07 |
| 86 | 97 | LELINGYIQKIK | 3 | 7.36 | 0.32 | 0.56 | 1.58 | 0.39 | 0.53 | 1.38 | 0.29 | 0.60 | 1.63 | 9.72 | 0.03 | 0.04 | 0.02 | 0.08 | 0.03 | 0.02 | 0.02 | 0.15 | 0.03 | 0.05 |
| 86 | 103 | LELINGYIQKIKSGEEDF | 3 | 8.09 | 2.63 | 4.71 | 6.48 | 2.57 | 4.69 | 6.17 | 2.56 | 4.92 | 6.57 | 14.93 | 0.04 | 0.24 | 0.02 | 0.10 | 0.05 | 0.06 | 0.06 | 0.07 | 0.03 | 0.12 |
| 87 | 95 | ELINGYIQK | 2 | 6.48 | 0.22 | 0.35 | 0.87 | 0.28 | 0.33 | 0.77 | 0.22 | 0.44 | 0.86 | 6.84 | 0.03 | 0.03 | 0.03 | 0.04 | 0.02 | 0.09 | 0.02 | 0.08 | 0.01 | 0.00 |
| 87 | 97 | ELINGYIQKIK | 3 | 7.29 | 0.28 | 0.59 | 1.48 | 0.34 | 0.57 | 1.20 | 0.26 | 0.71 | 1.58 | 8.89 | 0.00 | 0.12 | 0.14 | 0.04 | 0.07 | 0.08 | 0.03 | 0.14 | 0.06 | 0.07 |
| 87 | 102 | ELINGYIQKIKSGEED | 3 | 7.1 | 1.67 | 3.88 | 5.43 | 1.71 | 3.74 | 5.22 | 1.63 | 3.98 | 5.51 | 13.17 | 0.02 | 0.04 | 0.05 | 0.12 | 0.08 | 0.04 | 0.08 | 0.14 | 0.05 | 0.06 |
| 87 | 103 | ELINGYIQKIKSGEEDF | 3 | 7.78 | 2.61 | 4.94 | 6.53 | 2.57 | 4.74 | 6.22 | 2.55 | 4.91 | 6.53 | 14.13 | 0.06 | 0.03 | 0.02 | 0.09 | 0.06 | 0.08 | 0.08 | 0.18 | 0.07 | 0.06 |
| 88 | 95 | LINGYIQK | 2 | 6.06 | 0.21 | 0.34 | 0.79 | 0.27 | 0.30 | 0.69 | 0.20 | 0.40 | 0.84 | 5.81 | 0.02 | 0.00 | 0.03 | 0.06 | 0.02 | 0.01 | 0.01 | 0.07 | 0.02 | 0.00 |
| 88 | 97 | LINGYIQKIK | 3 | 6.43 | 0.26 | 0.54 | 1.37 | 0.32 | 0.47 | 1.22 | 0.26 | 0.61 | 1.44 | 6.98 | 0.02 | 0.03 | 0.02 | 0.05 | 0.04 | 0.02 | 0.01 | 0.07 | 0.05 | 0.06 |
| 88 | 103 | LINGYIQKIKSGEEDF | 3 | 7.33 | 2.60 | 4.79 | 6.28 | 2.52 | 4.71 | 6.07 | 2.55 | 4.88 | 6.32 | 12.03 | 0.06 | 0.09 | 0.02 | 0.07 | 0.07 | 0.06 | 0.07 | 0.14 | 0.02 | 0.05 |
| 89 | 95 | INGYIQK | 2 | 5.49 | 0.20 | 0.33 | 0.75 | 0.22 | 0.29 | 0.61 | 0.16 | 0.36 | 0.76 | 5.05 | 0.02 | 0.02 | 0.02 | 0.03 | 0.01 | 0.02 | 0.03 | 0.04 | 0.02 | 0.02 |
| 89 | 97 | INGYIQKIK | 3 | 5.89 | 0.29 | 0.57 | 1.44 | 0.29 | 0.48 | 1.26 | 0.29 | 0.67 | 1.49 | 6.78 | 0.02 | 0.03 | 0.03 | 0.02 | 0.03 | 0.01 | 0.02 | 0.07 | 0.04 | 0.11 |
| 89 | 101 | INGYIQKIKSGEE | 3 | 6.16 | 1.24 | 3.00 | 4.51 | 1.24 | 3.02 | 4.43 | 1.22 | 3.21 | 4.59 | 10.13 | 0.09 | 0.17 | 0.09 | 0.13 | 0.13 | 0.13 | 0.07 | 0.11 | 0.06 | 0.03 |
| 89 | 102 | INGYIQKIKSGEED | 2 | 6.1 | 1.78 | 3.95 | 5.36 | 1.77 | 3.81 | 5.08 | 1.75 | 4.01 | 5.35 | 10.99 | 0.04 | 0.09 | 0.05 | 0.10 | 0.05 | 0.04 | 0.07 | 0.16 | 0.08 | 0.04 |
| 89 | 103 | INGYIQKIKSGEEDF | 3 | 6.96 | 2.69 | 4.92 | 6.35 | 2.63 | 4.80 | 6.15 | 2.65 | 4.96 | 6.38 | 11.94 | 0.05 | 0.07 | 0.03 | 0.08 | 0.07 | 0.05 | 0.07 | 0.15 | 0.03 | 0.04 |
| 90 | 97 | NGYIQKIK | 2 | 6.81 | 0.26 | 0.43 | 1.29 | 0.28 | 0.38 | 1.12 | 0.20 | 0.50 | 1.36 | 7.41 | 0.02 | 0.00 | 0.01 | 0.07 | 0.01 | 0.02 | 0.02 | 0.09 | 0.01 | 0.02 |
| 90 | 103 | NGYIQKIKSGEEDF | 2 | 7 | 3.75 | 5.46 | 6.39 | 3.81 | 5.44 | 6.34 | 3.77 | 5.55 | 6.54 | 9.67 | 0.05 | 0.14 | 0.07 | 0.06 | 0.11 | 0.12 | 0.04 | 0.18 | 0.12 | 0.13 |
| 92 | 103 | YIQKIKSGEEDF | 3 | 6.23 | 2.30 | 4.20 | 5.50 | 2.25 | 4.18 | 5.40 | 2.28 | 4.22 | 5.56 | 8.69 | 0.04 | 0.13 | 0.03 | 0.07 | 0.08 | 0.06 | 0.05 | 0.14 | 0.05 | 0.08 |
| 93 | 102 | IQKIKSGEED | 3 | 5.02 | 1.52 | 3.53 | 4.73 | 1.50 | 3.44 | 4.60 | 1.48 | 3.51 | 4.78 | 7.52 | 0.04 | 0.14 | 0.05 | 0.06 | 0.09 | 0.09 | 0.05 | 0.15 | 0.06 | 0.06 |
| 93 | 103 | IQKIKSGEEDF | 2 | 6.02 | 2.19 | 4.03 | 5.17 | 2.18 | 4.07 | 5.17 | 2.19 | 4.03 | 5.27 | 7.80 | 0.02 | 0.13 | 0.06 | 0.06 | 0.06 | 0.05 | 0.07 | 0.13 | 0.08 | 0.09 |
| 107 | 125 | ASQFSDCSSAKARGDLGAF | 3 | 6.92 | 4.64 | 7.22 | 9.33 | 4.59 | 7.09 | 9.30 | 4.52 | 7.41 | 9.62 | 13.51 | 0.23 | 0.23 | 0.03 | 0.10 | 0.09 | 0.18 | 0.10 | 0.28 | 0.23 | 0.29 |
| 110 | 125 | FSDCSSAKARGDLGAF | 3 | 6.84 | 3.87 | 6.55 | 8.25 | 4.00 | 6.65 | 8.44 | 3.95 | 6.75 | 8.55 | 10.94 | 0.07 | 0.20 | 0.06 | 0.07 | 0.14 | 0.14 | 0.12 | 0.28 | 0.09 | 0.06 |
| 111 | 124 | SDCSSAKARGDLGA | 3 | 5.75 | 4.30 | 6.65 | 8.04 | 4.43 | 6.77 | 8.29 | 4.26 | 6.63 | 8.21 | 10.73 | 0.09 | 0.27 | 0.15 | 0.08 | 0.07 | 0.07 | 0.08 | 0.34 | 0.13 | 0.04 |
| 111 | 125 | SDCSSAKARGDLGAF | 3 | 5.73 | 4.17 | 6.54 | 7.93 | 4.23 | 6.59 | 8.17 | 4.11 | 6.58 | 8.18 | 10.58 | 0.06 | 0.28 | 0.06 | 0.22 | 0.08 | 0.10 | 0.04 | 0.33 | 0.11 | 0.04 |
| 113 | 122 | CSSAKARGDL | 2 | 5.17 | 2.68 | 4.22 | 5.20 | 2.71 | 4.36 | 5.25 | 2.64 | 4.18 | 5.25 | 7.54 | 0.02 | 0.15 | 0.07 | 0.04 | 0.10 | 0.14 | 0.12 | 0.23 | 0.03 | 0.19 |
| 113 | 125 | CSSAKARGDLGAF | 3 | 6.4 | 3.02 | 5.65 | 7.19 | 3.13 | 5.65 | 7.43 | 3.03 | 5.74 | 7.34 | 9.44 | 0.03 | 0.18 | 0.07 | 0.09 | 0.13 | 0.10 | 0.06 | 0.26 | 0.09 | 0.05 |
| 114 | 122 | SSAKARGDL | 2 | 5.07 | 2.29 | 3.47 | 4.48 | 2.28 | 3.60 | 4.58 | 2.31 | 3.73 | 4.65 | 6.64 | 0.15 | 0.07 | 0.06 | 0.15 | 0.07 | 0.03 | 0.06 | 0.24 | 0.07 | 0.03 |
| 114 | 132 | SSAKARGDLGAFSRGQMQK | 4 | 5.75 | 7.32 | 11.73 | 13.06 | 7.16 | 11.43 | 13.00 | 7.37 | 11.55 | 13.02 | 16.00 | 0.01 | 0.21 | 0.07 | 0.08 | 0.17 | 0.20 | 0.11 | 0.20 | 0.26 | 0.06 |
| 115 | 125 | SAKARGDLGAF | 2 | 6.37 | 2.49 | 4.75 | 6.05 | 2.53 | 4.86 | 6.23 | 2.53 | 4.81 | 6.13 | 8.01 | 0.08 | 0.17 | 0.07 | 0.14 | 0.07 | 0.12 | 0.14 | 0.22 | 0.09 | 0.06 |
| 118 | 138 | ARGDLGAFSRGQMQKPFEDAS | 4 | 6.7 | 5.59 | 9.07 | 11.16 | 5.50 | 8.94 | 11.02 | 5.55 | 9.16 | 11.20 | 14.56 | 0.06 | 0.32 | 0.02 | 0.07 | 0.18 | 0.15 | 0.05 | 0.43 | 0.19 | 0.33 |
| 123 | 138 | GAFSRGQMQKPFEDAS | 2 | 6.43 | 4.96 | 7.67 | 9.27 | 4.82 | 7.71 | 8.94 | 5.10 | 7.89 | 9.41 | 12.06 | 0.06 | 0.28 | 0.13 | 0.06 | 0.17 | 0.22 | 0.08 | 0.26 | 0.21 | 0.02 |
| 123 | 139 | GAFSRGQMQKPFEDASF | 2 | 7.24 | 4.79 | 7.58 | 8.93 | 4.78 | 7.41 | 8.77 | 4.80 | 7.63 | 8.96 | 12.32 | 0.05 | 0.24 | 0.05 | 0.05 | 0.21 | 0.19 | 0.04 | 0.26 | 0.22 | 0.15 |
| 125 | 139 | FSRGQMQKPFEDASF | 3 | 7.11 | 4.55 | 6.33 | 7.32 | 4.52 | 6.29 | 7.19 | 4.53 | 6.40 | 7.41 | 10.63 | 0.08 | 0.13 | 0.03 | 0.05 | 0.16 | 0.15 | 0.04 | 0.19 | 0.12 | 0.09 |
| 126 | 136 | SRGQMQKPFED | 2 | 5.78 | 3.97 | 5.68 | 6.31 | 3.94 | 5.76 | 6.41 | 3.95 | 5.65 | 6.34 | 7.54 | 0.06 | 0.18 | 0.04 | 0.03 | 0.05 | 0.07 | 0.07 | 0.21 | 0.09 | 0.01 |
| 126 | 138 | SRGQMQKPFEDAS | 2 | 5.91 | 3.84 | 5.72 | 6.60 | 3.92 | 5.77 | 6.69 | 3.80 | 5.65 | 6.72 | 9.32 | 0.04 | 0.12 | 0.04 | 0.09 | 0.05 | 0.03 | 0.08 | 0.14 | 0.06 | 0.12 |
| 126 | 139 | SRGQMQKPFEDASF | 3 | 6.88 | 3.76 | 5.46 | 6.36 | 3.80 | 5.42 | 6.28 | 3.75 | 5.53 | 6.50 | 9.62 | 0.04 | 0.14 | 0.07 | 0.06 | 0.12 | 0.13 | 0.04 | 0.19 | 0.11 | 0.09 |
| 129 | 139 | QMQKPFEDASF | 2 | 7.2 | 2.35 | 3.29 | 4.12 | 2.39 | 3.20 | 4.02 | 2.31 | 3.34 | 4.12 | 7.18 | 0.07 | 0.04 | 0.01 | 0.09 | 0.08 | 0.09 | 0.02 | 0.18 | 0.14 | 0.03 |
| 139 | 151 | FALRTGEMSGPVF | 2 | 7.84 | 2.40 | 4.19 | 5.55 | 2.49 | 4.19 | 5.53 | 2.39 | 4.26 | 5.62 | 9.37 | 0.02 | 0.04 | 0.01 | 0.06 | 0.05 | 0.02 | 0.07 | 0.16 | 0.06 | 0.07 |
| 139 | 157 | FALRTGEMSGPVFTDSGIH | 3 | 7.43 | 3.20 | 5.20 | 6.98 | 3.36 | 5.14 | 6.90 | 3.18 | 5.32 | 7.13 | 13.52 | 0.04 | 0.06 | 0.05 | 0.12 | 0.23 | 0.21 | 0.07 | 0.19 | 0.21 | 0.12 |
| 140 | 145 | ALRTGE | 2 | 4.98 | 1.43 | 1.70 | 2.60 | 1.53 | 1.71 | 2.54 | 1.45 | 1.75 | 2.64 | 4.35 | 0.01 | 0.08 | 0.03 | 0.07 | 0.02 | 0.01 | 0.02 | 0.14 | 0.03 | 0.02 |
| 140 | 146 | ALRTGEM | 2 | 5.71 | 1.35 | 2.06 | 3.30 | 1.48 | 2.09 | 3.26 | 1.36 | 2.13 | 3.35 | 4.82 | 0.01 | 0.10 | 0.04 | 0.07 | 0.06 | 0.07 | 0.02 | 0.16 | 0.04 | 0.06 |
| 140 | 148 | ALRTGEMSG | 2 | 5.42 | 1.90 | 3.30 | 4.79 | 2.07 | 3.38 | 4.86 | 1.90 | 3.33 | 4.85 | 7.60 | 0.03 | 0.08 | 0.07 | 0.09 | 0.10 | 0.19 | 0.06 | 0.19 | 0.08 | 0.10 |
| 140 | 151 | ALRTGEMSGPVF | 2 | 7.23 | 2.14 | 3.83 | 4.94 | 2.27 | 3.85 | 4.99 | 2.15 | 3.90 | 5.03 | 8.13 | 0.02 | 0.09 | 0.04 | 0.06 | 0.07 | 0.08 | 0.05 | 0.15 | 0.09 | 0.11 |
| 140 | 156 | ALRTGEMSGPVFTDSGI | 3 | 7.56 | 3.10 | 4.73 | 6.41 | 3.05 | 4.73 | 6.28 | 3.09 | 4.89 | 6.42 | 11.24 | 0.05 | 0.08 | 0.05 | 0.13 | 0.23 | 0.21 | 0.06 | 0.16 | 0.14 | 0.11 |
| 140 | 157 | ALRTGEMSGPVFTDSGIH | 2 | 6.9 | 3.38 | 5.24 | 6.90 | 3.57 | 5.27 | 6.91 | 3.37 | 5.35 | 7.04 | 13.02 | 0.02 | 0.21 | 0.07 | 0.11 | 0.22 | 0.21 | 0.07 | 0.23 | 0.21 | 0.05 |

| | | | | | | | | | | | | | | | | | | | | | | | | |
|---|---|---|---|---|---|---|---|---|---|---|---|---|---|---|---|---|---|---|---|---|---|---|---|---|
| 140 | 159 | ALRTGEMSGPVFTDSGIHII | 3 | 7.86 | 3.22 | 5.05 | 6.57 | 3.37 | 5.00 | 6.57 | 3.12 | 5.11 | 6.65 | 14.03 | 0.17 | 0.12 | 0.03 | 0.13 | 0.20 | 0.19 | 0.09 | 0.23 | 0.19 | 0.22 |
| 140 | 160 | ALRTGEMSGPVFTDSGIHIIL | 3 | 8.31 | 3.25 | 4.97 | 6.72 | 3.39 | 5.05 | 6.66 | 3.24 | 5.08 | 6.84 | 15.27 | 0.03 | 0.33 | 0.06 | 0.10 | 0.14 | 0.17 | 0.05 | 0.41 | 0.13 | 0.18 |
| 141 | 151 | LRTGEMSGPVF | 2 | 7.29 | 2.01 | 3.53 | 4.29 | 2.07 | 3.56 | 4.36 | 1.96 | 3.58 | 4.37 | 7.15 | 0.03 | 0.08 | 0.04 | 0.03 | 0.08 | 0.07 | 0.04 | 0.13 | 0.10 | 0.12 |
| 141 | 157 | LRTGEMSGPVFTDSGIH | 3 | 6.84 | 3.28 | 4.93 | 6.30 | 3.45 | 5.02 | 6.27 | 3.24 | 4.98 | 6.39 | 12.04 | 0.04 | 0.32 | 0.06 | 0.10 | 0.16 | 0.18 | 0.06 | 0.32 | 0.18 | 0.13 |
| 141 | 160 | LRTGEMSGPVFTDSGIHIIL | 3 | 8.23 | 3.20 | 4.79 | 6.25 | 3.31 | 4.90 | 6.23 | 3.15 | 4.91 | 6.31 | 14.73 | 0.04 | 0.35 | 0.05 | 0.13 | 0.13 | 0.14 | 0.05 | 0.41 | 0.10 | 0.14 |
| 142 | 157 | RTGEMSGPVFTDSGIH | 2 | 6.73 | 2.51 | 4.20 | 5.38 | 2.66 | 4.30 | 5.53 | 2.53 | 4.30 | 5.47 | 10.94 | 0.04 | 0.10 | 0.10 | 0.05 | 0.06 | 0.13 | 0.07 | 0.27 | 0.21 | 0.11 |
| 142 | 160 | RTGEMSGPVFTDSGIHIIL | 3 | 8.26 | 2.56 | 4.10 | 5.30 | 2.60 | 4.12 | 5.28 | 2.44 | 4.16 | 5.38 | 13.45 | 0.03 | 0.30 | 0.06 | 0.10 | 0.08 | 0.12 | 0.07 | 0.39 | 0.08 | 0.16 |
| 143 | 153 | TGEMSGPVFTD | 2 | 6.93 | 1.53 | 2.73 | 3.04 | 1.51 | 2.80 | 3.12 | 1.56 | 2.74 | 3.16 | 5.70 | 0.05 | 0.12 | 0.02 | 0.04 | 0.07 | 0.06 | 0.04 | 0.18 | 0.05 | 0.04 |
| 143 | 155 | TGEMSGPVFTDSG | 2 | 6.85 | 3.27 | 4.48 | 5.15 | 3.12 | 4.12 | 4.81 | 3.40 | 4.53 | 5.24 | 9.31 | 0.00 | 0.24 | 0.07 | 0.15 | 0.08 | 0.13 | 0.08 | 0.21 | 0.06 | 0.05 |
| 143 | 156 | TGEMSGPVFTDSGI | 2 | 7.69 | 2.41 | 3.87 | 4.71 | 2.44 | 3.87 | 4.67 | 2.42 | 3.90 | 4.76 | 8.59 | 0.01 | 0.18 | 0.05 | 0.04 | 0.16 | 0.16 | 0.03 | 0.14 | 0.14 | 0.08 |
| 143 | 157 | TGEMSGPVFTDSGIH | 2 | 7.05 | 2.60 | 4.12 | 5.13 | 2.69 | 4.21 | 5.14 | 2.59 | 4.14 | 5.17 | 10.09 | 0.02 | 0.26 | 0.09 | 0.07 | 0.10 | 0.06 | 0.04 | 0.29 | 0.10 | 0.07 |
| 143 | 159 | TGEMSGPVFTDSGIHII | 2 | 8.14 | 2.26 | 4.02 | 4.69 | 2.39 | 3.91 | 4.75 | 2.28 | 4.18 | 4.78 | 10.83 | 0.02 | 0.12 | 0.04 | 0.07 | 0.20 | 0.14 | 0.03 | 0.21 | 0.11 | 0.21 |
| 143 | 160 | TGEMSGPVFTDSGIHIIL | 2 | 8.31 | 2.50 | 3.90 | 4.93 | 2.59 | 4.05 | 4.97 | 2.48 | 4.03 | 4.74 | 12.87 | 0.02 | 0.28 | 0.03 | 0.09 | 0.08 | 0.09 | 0.05 | 0.41 | 0.20 | 0.13 |
| 144 | 160 | GEMSGPVFTDSGIHIIL | 2 | 8.61 | 2.37 | 3.79 | 4.68 | 2.38 | 3.68 | 4.76 | 2.33 | 3.79 | 4.67 | 12.78 | 0.02 | 0.35 | 0.03 | 0.19 | 0.17 | 0.08 | 0.06 | 0.45 | 0.02 | 0.25 |
| 145 | 157 | EMSGPVFTDSGIH | 2 | 7.01 | 2.49 | 3.57 | 4.25 | 2.50 | 3.67 | 4.23 | 2.43 | 3.75 | 4.26 | 9.11 | 0.03 | 0.22 | 0.03 | 0.04 | 0.09 | 0.09 | 0.04 | 0.08 | 0.06 | 0.04 |
| 145 | 160 | EMSGPVFTDSGIHIIL | 2 | 8.53 | 2.26 | 3.21 | 4.03 | 2.35 | 3.38 | 4.06 | 2.20 | 3.23 | 3.84 | 11.84 | 0.04 | 0.25 | 0.06 | 0.14 | 0.05 | 0.02 | 0.03 | 0.43 | 0.25 | 0.14 |
| 146 | 160 | MSGPVFTDSGIHIIL | 2 | 8.32 | 2.30 | 3.33 | 3.99 | 2.34 | 3.43 | 4.01 | 2.28 | 3.39 | 4.00 | 11.06 | 0.02 | 0.24 | 0.03 | 0.05 | 0.07 | 0.08 | 0.04 | 0.29 | 0.05 | 0.09 |
| 147 | 157 | SGPVFTDSGIH | 2 | 6.48 | 2.19 | 2.90 | 3.53 | 2.30 | 3.07 | 3.63 | 2.20 | 2.93 | 3.65 | 7.73 | 0.00 | 0.27 | 0.06 | 0.04 | 0.06 | 0.07 | 0.03 | 0.26 | 0.10 | 0.09 |
| 147 | 160 | SGPVFTDSGIHIIL | 2 | 8.27 | 1.98 | 2.60 | 3.24 | 2.05 | 2.67 | 3.24 | 1.98 | 2.66 | 3.29 | 10.20 | 0.01 | 0.23 | 0.03 | 0.06 | 0.08 | 0.09 | 0.03 | 0.26 | 0.06 | 0.08 |
| 149 | 157 | PVFTDSGIH | 2 | 6.45 | 2.10 | 2.73 | 3.27 | 2.11 | 2.77 | 3.32 | 2.18 | 2.82 | 3.34 | 6.34 | 0.04 | 0.20 | 0.04 | 0.05 | 0.02 | 0.12 | 0.06 | 0.19 | 0.14 | 0.10 |
| 149 | 160 | PVFTDSGIHIIL | 2 | 8.43 | 2.08 | 2.69 | 3.12 | 2.01 | 2.51 | 3.21 | 2.06 | 2.52 | 2.98 | 9.04 | 0.14 | 0.12 | 0.07 | 0.06 | 0.04 | 0.22 | 0.14 | 0.20 | 0.03 | 0.07 |
| 152 | 160 | TDSGIHIIL | 2 | 7.79 | 0.69 | 0.92 | 1.43 | 0.79 | 0.91 | 1.44 | 0.68 | 0.98 | 1.50 | 6.37 | 0.02 | 0.06 | 0.02 | 0.07 | 0.04 | 0.03 | 0.01 | 0.11 | 0.02 | 0.04 |
| 153 | 160 | DSGIHIIL | 2 | 7.76 | 0.26 | 0.49 | 0.95 | 0.34 | 0.45 | 0.95 | 0.22 | 0.56 | 0.99 | 5.89 | 0.02 | 0.00 | 0.01 | 0.08 | 0.04 | 0.02 | 0.01 | 0.11 | 0.03 | 0.01 |
| 154 | 160 | SGIHIIL | 2 | 7.71 | 0.18 | 0.21 | 0.26 | 0.24 | 0.20 | 0.30 | 0.16 | 0.26 | 0.31 | 5.05 | 0.01 | 0.00 | 0.02 | 0.05 | 0.01 | 0.01 | 0.01 | 0.06 | 0.01 | 0.02 |
